# Mutation of YFT3, an isomerase in the isoprenoid biosynthetic pathway, impairs its catalytic activity and carotenoid accumulation in tomato fruit

**DOI:** 10.1093/hr/uhae202

**Published:** 2024-07-24

**Authors:** Wenzhen Li, Lulu Chen, Weihua Zhao, Yuhang Li, Ying Chen, Tengjian Wen, Zhengjun Liu, Chao Huang, Lida Zhang, Lingxia Zhao

**Affiliations:** Department of Plant Science, School of Agriculture and Biology, Shanghai Jiao Tong University, 800 Dongchuan Road, Shanghai 200240, China; Joint Tomato Research Institute, School of Agriculture and Biology, Shanghai Jiao Tong University, 800 Dongchuan Road, Shanghai 200240, China; Department of Plant Science, School of Agriculture and Biology, Shanghai Jiao Tong University, 800 Dongchuan Road, Shanghai 200240, China; Jiangsu Key Laboratory for Bioresources of Saline Soils, Jiangsu Synthetic Innovation Center for Coastal Bio-agriculture, School of Wetland, Yancheng Teachers University, 2 South Xiwang Avenue, Yancheng 224002, China; Department of Plant Science, School of Agriculture and Biology, Shanghai Jiao Tong University, 800 Dongchuan Road, Shanghai 200240, China; Joint Tomato Research Institute, School of Agriculture and Biology, Shanghai Jiao Tong University, 800 Dongchuan Road, Shanghai 200240, China; Department of Plant Science, School of Agriculture and Biology, Shanghai Jiao Tong University, 800 Dongchuan Road, Shanghai 200240, China; Joint Tomato Research Institute, School of Agriculture and Biology, Shanghai Jiao Tong University, 800 Dongchuan Road, Shanghai 200240, China; Youlaigu Science and Technology Innovation Center, 588 West Chenfeng, Yushan town, Agriculture Service Center, Kunshan 215300, China; Department of Plant Science, School of Agriculture and Biology, Shanghai Jiao Tong University, 800 Dongchuan Road, Shanghai 200240, China; Joint Tomato Research Institute, School of Agriculture and Biology, Shanghai Jiao Tong University, 800 Dongchuan Road, Shanghai 200240, China; National Key Laboratory of Green Pesticide, Key Laboratory of Green Pesticide and Agricultural Bioengineering, Ministry of Education, Guizhou University, 2708 South Huaxi Avenue, Guiyang 550025, China; Zhejiang Provincial Key TCM Laboratory for Chinese Resource Innovation and Transformation, College of Pharmaceutical Science, Zhejiang Chinese Medical University, 548 Binwen Road, Hangzhou 310053, China; Department of Plant Science, School of Agriculture and Biology, Shanghai Jiao Tong University, 800 Dongchuan Road, Shanghai 200240, China; Joint Tomato Research Institute, School of Agriculture and Biology, Shanghai Jiao Tong University, 800 Dongchuan Road, Shanghai 200240, China; Department of Plant Science, School of Agriculture and Biology, Shanghai Jiao Tong University, 800 Dongchuan Road, Shanghai 200240, China; Joint Tomato Research Institute, School of Agriculture and Biology, Shanghai Jiao Tong University, 800 Dongchuan Road, Shanghai 200240, China

## Abstract

Tomato fruit colors are directly associated with their appearance quality and nutritional value. However, tomato fruit color formation is an intricate biological process that remains elusive. In this work we characterized a tomato *yellow fruited tomato 3* (*yft3*, *e9292*, *Solanum lycopersicum*) mutant with yellow fruits. By the map-based cloning approach, we identified a transversion mutation (A2117C) in the *YFT3* gene encoding a putative isopentenyl diphosphate isomerase (SlIDI1) enzyme, which may function in the isoprenoid biosynthetic pathway by catalyzing conversion between isopentenyl pyrophosphate (IPP) and dimethylallyl pyrophosphate (DMAPP). The mutated *YFT3* (A2117C) (designated *YFT3 allele*) and the *YFT3* genes did not show expression difference at protein level, and their encoded YFT3 allelic (S126R) and YFT3 proteins were both localized in plastids. However, the transcript levels of eight genes (*DXR*, *DXS*, *HDR*, *PSY1*, *CRTISO, CYCB*, *CYP97A*, and *NCED*) associated with carotenoid synthesis were upregulated in fruits of both *yft3* and *YFT3* knockout (*YFT3-KO*) lines at 35 and 47 days post-anthesis compared with the red-fruit tomato cultivar (M82). *In vitro* and *in vivo* biochemical analyses indicated that YFT3 (S126R) possessed much lower enzymatic activities than the YFT3 protein, indicating that the S126R mutation can impair YFT3 activity. Molecular docking analysis showed that the YFT3 allele has higher ability to recruit isopentenyl pyrophosphate (IPP), but abolishes attachment of the Mg^2+^ cofactor to IPP, suggesting that Ser126 is a critical residue for YTF3 biochemical and physiological functions. As a result, the *yft3* mutant tomato line has low carotenoid accumulation and abnormal chromoplast development, which results in yellow ripe fruits. This study provides new insights into molecular mechanisms of tomato fruit color formation and development.

## Introduction

The coloration of many ripe fleshy fruits is determined by the composition and accumulation of carotenoid pigments with 40-carbon hydrocarbons, which can be categorized as carotenes and xanthophylls [1], in addition to yellow, orange, and red isoprenoid pigments [2]. Carotenoids promote light harvesting as accessory pigments in photosynthesis, protect the photosynthetic apparatus from photo-oxidative damage, and accumulate in flowers and fruits to attract animals and insects, thereby facilitating the dispersal of pollen and seeds [1, 3–5]. They are also precursors of phytohormones such as abscisic acid (ABA), strigolactones (SLs), and other signaling molecules that are important for development and stress responses [2, 6]. Carotenoids and their derivatives are antioxidants and essential components of the human diet. For example, β-carotene, α-carotene, and zeaxanthin serve as the precursors for vitamin A biosynthesis. All-*trans*-lycopene has been associated with a reduced risk of cancer and cardiovascular disease [1–3, 6]. In addition to their functions as pigments and nutrients, carotenoids are precursors of volatile organic compounds released during fruit ripening [2, 7–9], conferring aromas attracting consumers [1, 10].

The tomato (*Solanum lycopersicum*) fruit has been widely used as a model system to dissect the molecular pathways that give rise to color variants and pigment syntheses. Examples include *psy1* (*r*) [11, 12], *slidi1* [2, 4], *Delta* [13], *Beta*, and *old-gold* [14], as well as *tangerine* [15]. The phytohormone ethylene participates in regulation of ripening in climacteric fruits, such as tomato, and plays a central role in regulating fruit coloration [16–18]. A number of transcription factors or signaling components, e.g. RIN (RIPENING INHIBTOR, MADS-box) [19, 20], CNR (COLORLESS NON RIPENING, Squamosa promoter-binding protein-like, SPB-box) [21], NOR (NONRIPENING) [22], TAG1 (TOMATO AGAMOUS-LIKE 1) [23], FRUITFULL 1 and 2, ETHYLENE RECEPTOR 3 (ETR3/NR) [24–26], SlHB1 (a tomato HD-ZIP homeobox protein) [27], SlAP2a [28], and WRKY 32 [29], as well as *YFT1* (YELLOW FRUITED TOMATO1) [17] and Never ripe (Nr) [30], have been revealed indirectly to be associated with fruit coloring and ethylene syntheses.

In the current study, we revealed the genetic basis of yellow coloration in the fruit of the *yellow fruited tomato 3* (*yft3*) tomato mutant. The single recessive *yft3* gene was successfully isolated using map-based cloning, and found to encode an isopentenyl diphosphate (IDI) enzyme. Carotenoids are a class of isoprene derivatives/isoprenoids, which are derived from C5 building blocks,IPP, and its isomer, dimethylallyl diphosphate (DMAPP). The interconversion between IPP and DMAPP is dependent on IDI [31, 32]. Some bacteria, as well as all vascular plants, have two distinct pathways for producing IPP and DMAPP, the cytoplasmic mevalonic acid (MVA) pathway and the plastidial 2-*C*-methyl-d-erythritol-4-phosphate (MEP) pathway [31, 33, 34]. The MVA pathway only produces IPP, as a substrate, which can be isomerized to DMAPP by IDI, implying that IDI is essential for the synthesis of DMAPP from IPP in eukaryotic organisms, such as in mitochondria, peroxisomes, and endoplasmic reticulum. However, the MEP pathway within plastids can contribute to both IPP and DMAPP derived from 4-hydroxy-3-methylbut-2-enyl diphosphate (HMBPP) by HMBPP reductase (HDR) at a ratio of 6:1 in the last step [31, 32, 35–37]. IDI functions to keep an appropriate ratio of IPP to DMAPP in plastids. However, since it exists in both the MVA and MEP pathways, IDI plays a key role in modulating IPP and DMAPP levels for isoprenoid synthesis in multiple subcellular compartments [4, 38].

Isoprenoid synthesis begins with head-to-tail condensation of DMAPP and IPP. DMAPP is extended by addition of IPP units to form short-chain prenyl diphosphates, such as geranyl diphosphate (GPP), farnesyl diphosphate (FPP), and geranylgeranyl diphosphate (GGPP) [32]. As an end-product in the MEP pathway, GGPP acts as an immediate precursor to produce the first C40 isoprenoid product phytoene in the carotenoid synthesis pathway (CSP) catalyzed by phytoene synthase (PSY) [3, 4]. In later-diverging land plants, as an immediate precursor of carotenogenesis, GGPP produced from the plastid MEP pathway can shuttle between the cytosol and plastids despite the IPP of the C5 building block being derived from both the MVA and MEP pathways [1]. Carotenoids in the chromoplasts of tomato fruit are exclusively produced via the MEP pathway, and the cytoplasmic IPP–DMAPP isomerization by IDI2 does not compensate for the loss of IDI1 activity in the plastids [4], suggesting that IDI1 activity is essential to avoid deficiency of DMAPP for carotenoid synthesis in plastids. However, the key amino acid residues for IDI1 function still remain elusive.

Here we report the genetic basis of yellow fruit in the tomato *yellow fruited tomato 3* (*yft3*) mutant. We mapped the single recessive *yft3* gene, whose wild-type (WT) allele encodes an IDI enzyme. We describe and discuss the molecular mechanism of how the S126R mutation in SlIDI1 imposes a major effect on SlIDI1 enzymatic activity and causes the yellow-colored tomato fruit phenotype in *yft3* mutant.

## Results

### A single recessive gene determines yellow-fruited phenotype in *yft3* mutant

The *yft3* (*e9292*) tomato line exhibited normal plant architecture and progression through fruit development (size and shape), and did not show apparent phenotypic differences other than its yellow fruit color from the WT (M82) with red fruit color at the ripening stage (54 days post-anthesis [dpa]) (Supplementary Data Fig. S1). We observed that all *F*_1_ hybrid progenies of *yft3* × M82 (5 plants) and *yft3* × LA1585 (18 plants) bore red color fruits but their *F*_2_ generation showed fruit color segregation at the ratio of 139/43 (red versus yellow) in *yft3* × M82 (χ^2^ = 0.183 < 3.84) and 91/25 (red versus yellow) in *yft3* × LA1585(χ^2^ = 0.736 < 3.84) (Table 1), sugesting that the yellow color of *yft3* fruit is caused by a single recessive gene at the *YFT3* allele.

**Table 1:** Segregation of fruit color in two genetic populations.

Population	Generation	Total plants	Plants bearing red fruit	Plants bearing yellow fruit	χ^2^ value^a^
*yft3* × M82	*F*_1_	5	5	0	
	*F*_2_	182	139	43	0.183
*yft3* × LA1585	*F*_1_	18	18	0	
	*F*_2_	116	91	25	0.736

a χ^2^ value (0.05, df = 1) = 3.84.

### Mapping of *YFT3* gene

**Figure 1:**
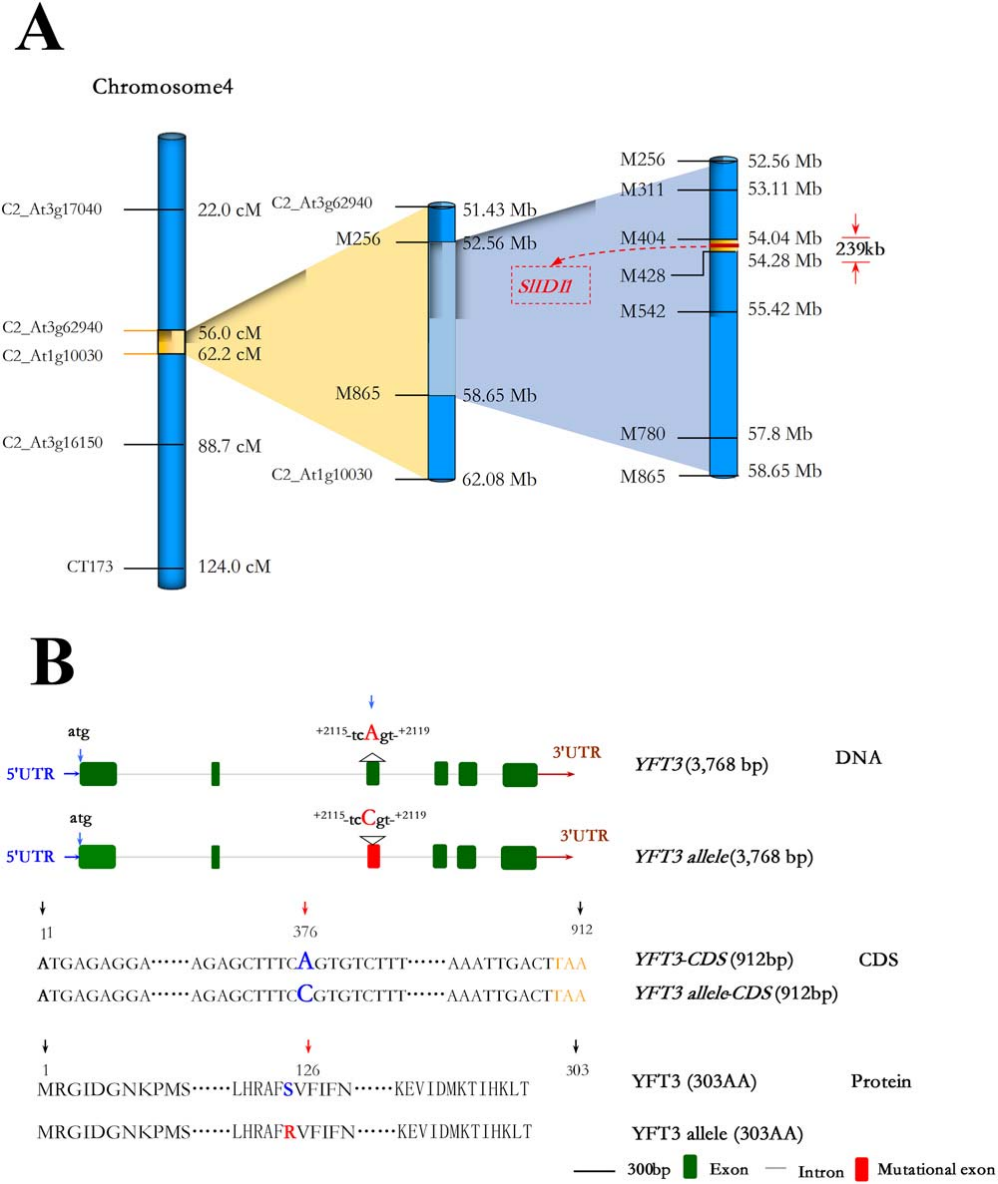
Map-based cloning of the *YFT3*/*YFT3* allele genes and the corresponding predicted protein sequences. **A** Mapping of *YFT3*. **B** Structural features of the *YFT3*/*YFT3* allele genes and the corresponding predicted protein sequences. ATG, start codon; CDS, coding sequence; TAA, stop codon; 5′UTR, 5′-untranslated region; 3′UTR, 3′-untranslated region; *YFT3*, a *yellow-fruited tomato 3* gene; its candidate gene is *SlIDI1*, and was isolated from the red-fruited M82 tomato; *YFT3 allele*, an allele of the *YFT3* gene, which carries a mutation at 2117 bp (A → C) downstream of the start codon ATG; it was isolated from the *yft3* mutant tomato.

Based on the fruit color of *F*_2_ individuals from the *yft3* × LA1585 cross (Supplementary Data Fig. S2), 116 plants were screened using 45 cleaved amplified polymorphic sequences (CAPS)/derived cleaved amplified polymorphic sequence (dCAPS) markers spanning all 12 tomato chromosomes (Supplementary Data Table S1). According to the fruit colors and genotypes, logarithm of the odds (LOD) scores were calculated by R/QTL analysis, and the maximal LOD score (24.10) was detected in a 10.65-Mb region between C2_At3g62940 (*SL2.50ch 04:51435545.0.51436456*) and C2_At1g10030 (*SL2.50ch04: 62086451.0.62088182*) of the CAPS markers on chromosome 4 (Supplementary Data Fig. S3), and *YFT3* was thereby mapped between C2_At3g62940 and C2_At1g10030 (Fig. 1A).

To further refine the position of the *YFT3* locus, an additional 1338 *yft3* × LA1585 *F*_2_ individuals were screened using seven new CAPS markers within the 10.65-Mb region. The fine mapping narrowed *YFT3* locus to a 239 330-bp region between the two CAPS markers M404 (*SL2.50ch4:54046020.0.54047002*) and M428 (*SL2.50ch4:54284003.0.54285299*); this region harbors 15 candidate genes (Fig. 1A, Supplementary Data Table S2). According to the tomato genome annotation (ITAG2.3, http://solgenomics.net), one of these 15 candidates, *Solyc04g056390* (named *SlIDI1*), encodes a putative isopentenyl diphosphate δ-isomerase, which acts as a catalytic enzyme in the interconversion of IPP and DAMPP in the MEP pathway [2, 4], and so directly affects the GGPP product and synthesis of carotenoid derivatives in the downstream CSP [31, 32]. Comparative analysis of genomic fragments amplified by PCR from M82 and *yft3* showed only a single base mutation (A → C, 54165646 bp) in the sequence of *YFT3*/*SlIDI1* (*Solyc04g056390*) in the *yft3* mutant (designated the *YFT3 allele*). The mutation is located at the third exon of the *YFT3 allele*, 2117 bp downstream of the ATG start codon (Fig. 1B), leading to an amino acid substitution (Ser126Arg) of the YFT3 allele protein in the *yft3* mutant (Fig. 1B).

Expression analysis showed that the *YFT3* gene was predominantly expressed in reproductive organs, such as flowers and fruits. The transcript level of the *YFT3* allele in *yft3* was higher than that of *YFT3* in all tested tissues except roots, stamens, and pistils in M82 (Supplementary Data Fig. S4).

### Functional complementation and loss of function assays for *YFT3* gene

**Figure 2:**
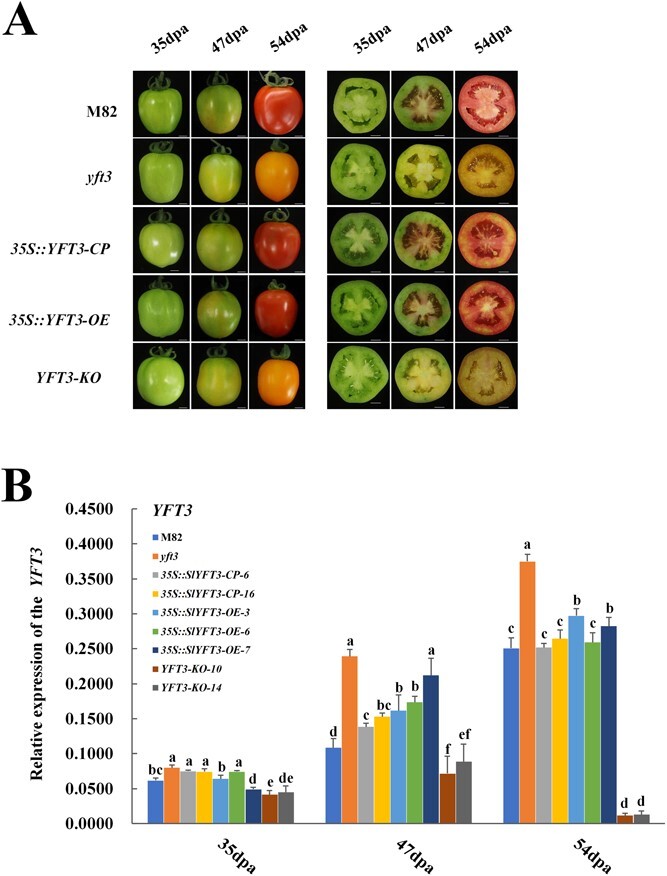
Functional complementation of *YFT3* in tomato. **A** Fruit colors of tomato lines with different genetic backgrounds at different developmental stages (scale bars = 1 cm). **B** Expression levels of *YFT3* in the fruit of tomato lines with different genetic backgrounds. 35 dpa, 47 dpa, and 54 dpa correspond to the mature green stage (MG), breaker stage (BR), and red/yellow ripening stage (RR/YR) during tomato fruit ripening. M82, wild type of the experimental material; *yft3*, *e9292* tomato mutant; *35S*::*YFT3-OE*, transgenic tomato lines created by transforming M82 with *35S*::*YFT3-CDS*; *35S::YFT3-CP,* transgenic tomato lines created by transforming *yft3* with *35S*:: *YFT3-CDS*; *YFT3-KO*, *YFT3* knockout lines created in M82 using CRISPR-cas9. Data indicate the mean ± standard deviation (*n* = 3). Lowercase letters indicate statistical significance at *P* < 0.05 as determined by Duncan’s test.

To determine where the *YFT3 allele* is responsible for the yellow-fruited phenotype in *yft3* mutant or not, we expressed the *YFT3* gene (*35S*::*YFT3-CP*) in the *yft3* mutant and observed that all the resulting transgenic *yft3* plants bore red fruits compared with the control with yellow fruits at fruit ripening stage (Fig. 2A). Intriguingly, when the *YFT3* gene in the M82 line was suppressed by expressing the *YFT3-KO* construct, its ripe fruits were changed from red to yellow (Fig. 2A), confirming that the *YFT3* gene plays a critical role in tomato fruit color formation.

*YFT3* expression in the *YFT3-KO-10/14* lines was significantly lower than in the red-fruited tomato lines, such as M82, 35S::*YFT3-CP-6/16*, and 35S::*YFT3-OE-3/6/7*, resulting in a yellow-fruited phenotype. However, fruits also had a yellow phenotype in *yft3* tomato with a high expression level of *YFT3* (Fig. 2A and B).

### Mutation in *YFT3 allele* affects chromoplast development

**Figure 3:**
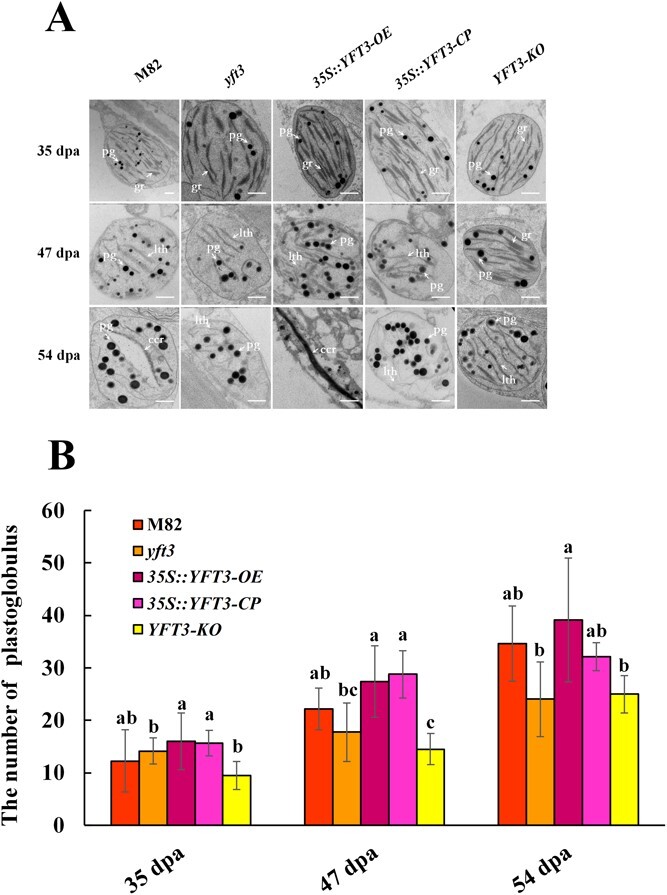
TEM imaging of chromoplast ultrastructure. **A** Chromoplast ultrastructure within pericarp cells among tomato lines with different genetic backgrounds at different stages. pg, plastoglobule; gr, grana; lth, long linear thylakoid membrane structure; ccr, carotenoid crystalloid. Scale bar = 500 nm. **B** Difference in plastoglobule number among different tomato lines at different stages. Data represent the mean values of three biological replicates. Eight different fields of view were observed for each biological repeat, and the error bars represent the standard deviations. Small letters indicate statistical significance in plastoglobule number at *P* < 0.05 as determined by Duncan’s test. M82, wild type of the experimental material; *yft3*, *e9292* tomato mutant; 35S::*YFT3-OE*, transgenic tomato lines created by transforming M82 with *35S*::*YFT3-CDS*; *35S::YFT3-CP*, transgenic tomato lines created by transforming *yft3* with *35S*:: *YFT3-CDS*; *YFT3-KO*, *YFT3* knockout in the M82 background, created using CRISPR-cas9. 35 dpa, 47 dpa, and 54 dpa correspond to the mature green stage (MG), breaker stage (BR), and red/yellow ripening stage (RR/YR) during tomato fruit ripening.

During tomato fruit ripening, chlorophyll is broken down and chloroplasts develop into chromoplasts along with carotenoid biosynthesis [17]. Consistently, we observed that the number of carotenoid-containing plastoglobules in fruit pericarp cells varied greatly among different tomato lines with fruit ripening (Fig. 3A and B). The number of plastoglobules in the yellow-fruited *yft3* and *YFT3-KO* lines was significantly lower than that in red-fruited M82, *YFT3-CDS-CP*, and *YFT3-CDS-OE* lines (Fig. 3A and B). In particular, in the M82 and *35S::YFT3-OE* lines, long strip-shaped crystalline bodies and/or undulating structures were observed in the chromoplasts with accumulation of carotenoids as ripening progressed, while these structures were rarely observed in *35S::YFT3-CP*, *yft3*, and *YFT3-KO* lines at 54 dpa (Fig. 3).

We also observed that the thylakoids, grana, and chloroplast envelope remained intact in the chloroplasts at 35 dpa, and there were no differences in chloroplast structure or integrity among *35S::YFT3-CP*, *35S::YFT3-OE*, *YFT3-KO*, *yft3*, and M82 lines (Fig. 3A). However, the chloroplast envelope and thylakoid structure began to degrade at 47 dpa in the *35S::YFT3-CP*, *35S::YFT3-OE*, and M82 lines; the linear thylakoid membrane could be visible at the same time point but had completely disappeared at 54 dpa. In contrast, distinguishable chloroplast envelopes and granular structures were still visible in both the *yft3* mutant and *YFT3-KO* lines at 47 and 54 dpa (Fig. 3A).

### *YFT3 allele* mutation affects expression of genes associated with carotenoid synthesis

**Figure 4:**
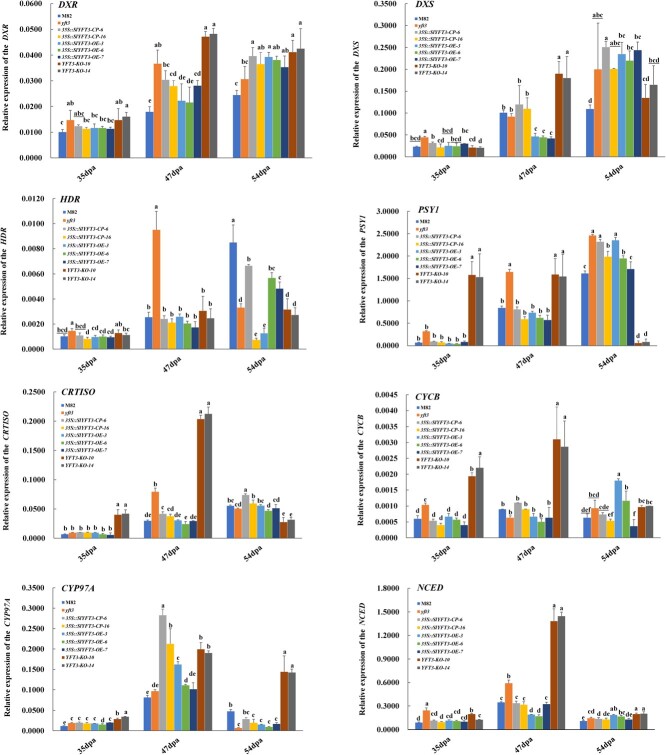
Expression of genes involved in the MEP pathway and CSP. *DXR*, 1-deoxy-d-xylulose 5-phosphate reductoisomerase; *DXS*, 1-deoxy-d-xylulose 5-phosphate synthase; *HDR*, 4-hydroxy-3-methylbut-2-enyl diphosphate reductase; *PSY1*, phytoene synthase 1; *CRTISO*, carotene isomerase; *CYCB*, lycopene β-cyclase; *CYP707A,* 8′-hydroxylase enzyme; *NCED*, 9-*cis*-epoxycarotenoid dioxygenases. M82, wild type of the experimental material; *yft3*, *e9292* tomato mutant created from M82 by chemical mutagenesis using EMS; 35S::*YFT3-OE*, transgenic tomato line created by transforming M82 with *35S*:: *YFT3-CDS*; *35S::YFT3-CP*, transgenic tomato line created by transforming *yft3* with *35S*:: *YFT3-CDS*; *YFT3-KO*, *YFT3* knockout line in the M82 background created using the CRISPR-cas9 technique. 35 dpa, 47 dpa, and 54 dpa correspond to the mature green stage (MG), breaker stage (BR), and red/yellow ripening stage (RR/YR) during tomato fruit ripening. Data are presented as mean ± standard deviation (*n* = 3). Lowercase letters indicate statistical differences between tomato lines tested using Duncan’s test at *P* < 0.05.

Carotenoid synthesis occurs within plastids via two successive pathways, MEP and CSP (Supplementary Data Fig. S5). We observed that, in general, transcript expression of genes involved in the MEP and CSP pathways, such as *1-DEOXY-D-XYLULOSE 5-PHOSPHATE REDUCTOISOMERASE* (*DXR*), *1-DEOXY-D-XYLULOSE 5-PHOSPHATE SYNTHASE* (*DXS*), *4-HYDROXY-3-METHYLBUT-2- ENYL DIPHOSPHATE REDUCTASE* (*HDR*), and *PHYTOENE SYNTHASE 1* (*PSY1*), *CAROTENE ISOMERASE* (*CRTISO*), increased with fruit ripening measured until 54 dpa in M82 (Fig. 4), whereas *LYCOPENE Β-CYCLASE* (*CYCB*), cytochrome P450-type monooxygenase 97A (*CYP97A*), and *9-CIS-EPOXYCAROTENOID DIOXYGENASES* (*NCEDs*) had higher expression level at 47 than at 54 dpa (Fig. 4).

There were no significant differences in the expression levels of eight carotenoid synthesis-associated genes (*DXR*, *DXS*, *HDR*, *PSY1*, *CRTISO*, *CYCB, CYP97A*, and *NCED*) among the red-fruited lines, including M82, *35S::YFT3-CP-6/16*, and *35S::YFT3-OE-3/6/7* at both 35 and 47 dpa*,* excluding *DXS* in *35S::YFT3-CP* lines at 47 dpa, and *CYP97A* in both *35S::YFT3-CP* and *35S::YFT3-OE* lines at 47 dpa. However, the mRNA levels of *DXR*, *DXS*, and *PSY1* in *35S::YFT3-CP* and *35S::YFT3-OE* lines were significantly higher than that of M82 at 54 dpa, whereas transcript expression of HDR and *CYP97A* in *35S::YFT3-CP* and *35S::YFT3-OE* lines was significantly lower than that of M82 at 54 dpa (Fig. 4).

In the yellow-fruited tomato lines of *yft3* and *YFT3-KO-10/14*, the expression levels of genes involved in the MEP and CSP pathways were higher than or close to that in M82 with the red-fruited phenotype during fruit ripening, but expressions of *HDR* and *CYP97A* in *yft3* and of *HDR*, *PSY1*, and *CRTISO* in *YFT3-KO* lines at 54 dpa were significantly lower than in M82 (Fig. 4).

The contents of lycopene, β-carotene, and total carotenoids were increased in the pericarp of all the tomato lines with fruit ripening, and were highest at 54 dpa (Table 2). The lycopene contents of *35S::YFT3-OE-3/6/7* were considerably higher than those in *35S::YFT3-CP-6/16* and M82 lines. In both *yft3* and *YFT3-KO-10/14* lines, lycopene contents were lower than those in *35S::YFT3-CP-6/16* and M82 lines. There were no significant differences in lycopene contents between the *35S::YFT3-CP-6/16* and M82 lines, *yft3* and *YFT3-KO-10/14* lines (Table 2). Furthermore, the levels of both β-carotene and total carotenoid showed the same patterns as lycopene. In red-fruited tomato lines, the ratio of lycopene to β-carotene was found to be significantly higher than in yellow-fruited lines. Specifically, the ratio was >10 in the red-fruited lines, such as *35S::YFT3-OE-3/6/7* (21.71, 27.63, and 29.21), *35S::YFT3-CP-6/16* (14.32 and 10.61), and M82 (14.56), whereas the ratios were less than or approximately 1 in the yellow-fruited lines like *YFT3-KO-10/14* (0.83 and 1.95) and *yft3* (0.74) (Table 2).

**Table 2:** Carotenoid content of fruit in tomato lines with different genetic backgrounds (μg$\square $g^−^1 FW).

	Lycopene	α-Carotene	β-Carotene	Lutein	Total carotenoids	Lycopene/β-carotene
35 dpa						
M82	n.d.	n.d.	1.50 ± 0.10 ^A^	4.50 ± 1.33 ^bc^	6.00 ± 1.42^ab^	
*yft3*	n.d.	n.d.	1.38 ± 0.09^A^	4.24 ± 1.17^c^	5.62 ± 1.10^b^	
*35S::YFT3-CP-6*	n.d.	n.d.	1.16 ± 0.25^AB^	6.29 ± 0.59^ab^	7.45 ± 0.77^ab^	
*35S::YFT3-CP-16*	n.d.	n.d.	1.12 ± 0.19^AB^	4.95 ± 0.90^abc^	6.07 ± 0.80^ab^	
*35S::YFT3-OE-3*	n.d.	n.d.	0.94 ± 0.14^B^	5.38 ± 0.07^abc^	6.32 ± 0.06^ab^	
*35S::YFT3-OE-6*	n.d.	n.d.	1.39 ± 0.12^A^	5.76 ± 0.62^abc^	7.14 ± 0.59^ab^	
*35S::YFT3-OE-7*	n.d.	n.d.	1.18 ± 0.25^AB^	6.56 ± 1.39^a^	7.74 ± 1.58^a^	
*YFT3-KO-10*	n.d.	n.d.	0.97 ± 0.11^B^	5.49 ± 1.87^abc^	6.46 ± 1.88^ab^	
*YFT3-KO-14*	n.d.	n.d.	1.32 ± 0.19^AB^	4.90 ± 0.78^abc^	6.22 ± 0.63^ab^	
47 dpa						
M82	1.67 ± 0.05^B^	n.d.	1.91 ± 0.27^BCD^	5.28 ± 0.80^ABC^	8.86 ± 0.67^ABC^	0.88
*yft3*	n.d.	n.d.	2.39 ± 0.34^AB^	7.86 ± 0.96^A^	10.25 ± 0.87^AB^	n.d.
*35S::YFT3-CP-6*	1.02 ± 0.02^C^	n.d.	1.92 ± 0.45^BCD^	7.31 ± 0.07^AB^	10.25 ± 0.13^AB^	0.53
*35S::YFT3-CP-16*	1.32 ± 0.20^BC^	n.d.	1.71 ± 0.03^CD^	4.21 ± 0.67^C^	7.23 ± 0.49^C^	0.77
*35S::YFT3-OE-3*	1.61 ± 0.47^BC^	n.d.	2.21 ± 0.43^ABC^	5.36 ± 1.25^ABC^	9.18 ± 2.12^ABC^	0.73
*35S::YFT3-OE-6*	1.78 ± 0.06^B^	n.d.	1.94 ± 0.19^BCD^	4.72 ± 1.02^C^	8.44 ± 0.82^BC^	0.92
*35S::YFT3-OE-7*	4.28 ± 0.59^A^	n.d.	2.58 ± 0.24^A^	4.87 ± 0.55^BC^	11.74 ± 1.38^A^	1.66
*YFT3-KO-10*	n.d.	n.d.	1.59 ± 0.07^D^	6.67 ± 0.52^ABC^	8.27 ± 0.60^BC^	n.d.
*YFT3-KO-14*	n.d.	n.d.	1.99 ± 0.11^BCD^	6.74 ± 2.39^ABC^	8.73 ± 2.29^BC^	n.d.
54 dpa						
M82	88.94 ± 6.94^B^	1.15 ± 0.28 ^CD^	6.11 ± 0.26^A^	6.83 ± 1.21^AB^	103.04 ± 8.06^B^	14.56
*yft3*	2.24 ± 0.53^D^	n.d.	3.02 ± 0.35^E^	6.68 ± 0.65^AB^	11.94 ± 1.13^D^	0.74
*35S::YFT3-CP-6*	77.26 ± 4.17^B^	0.81 ± 0.11 ^D^	5.40 ± 0.43^AB^	7.77 ± 1.53^A^	91.24 ± 4.21^B^	14.32
*35S::YFT3-CP-16*	47.23 ± 3.02^C^	0.28 ± 0.04^E^	4.45 ± 0.27^BCD^	6.64 ± 0.86^AB^	58.59 ± 2.96^C^	10.61
*35 S::YFT3-OE-3*	137.42 ± 9.50^A^	1.53 ± 0.34^BC^	6.33 ± 0.11^A^	7.44 ± 0.01^AB^	152.71 ± 9.37^A^	21.71
*35S::YFT3-OE-6*	139.95 ± 21.00^A^	1.59 ± 0.23^B^	5.07 ± 1.08^BC^	5.41 ± 0.85^BC^	152.01 ± 22.30^A^	27.63
*35S::YFT3-OE-7*	125.99 ± 1.82^A^	2.38 ± 0.15^A^	4.31 ± 0.09^CD^	6.59 ± 0.31^ABC^	139.27 ± 2.26^A^	29.21
*YFT3-KO-10*	3.09 ± 0.28^D^	n.d.	3.73 ± 0.23^DE^	6.32 ± 0.85^ABC^	13.15 ± 1.01^D^	0.83
*YFT3-KO-17*	2.97 ± 0.08^D^	n.d.	2.82 ± 0.25^E^	4.50 ± 0.81^C^	10.29 ± 1.06^D^	1.05

Data are mean ± standard deviation of the three biological replicates. Capital and small letters indicate statistical significance at *P* < 0.01 and *P* < 0.05, respectively, using Duncan’s test. n.d., not determined.

### Ser126Arg mutation does not alter subcellular localization and YFT3 allele protein levels

**Figure 5:**
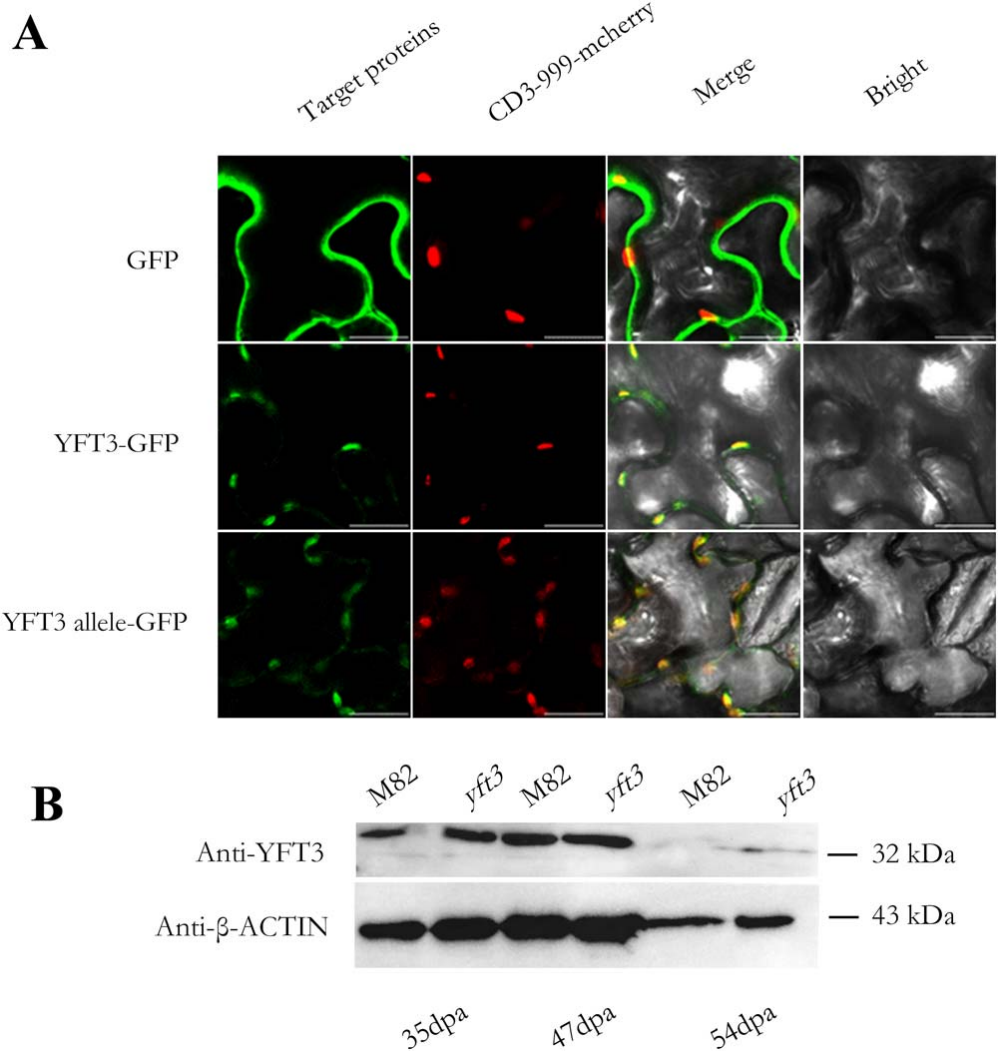
Subcellular localization and protein content of YFT3 and YFT3 allele determined by confocal imaging (**A**) and western blotting (**B**). Scale bars: 20 μm. M82, wild type of the experimental material; *yft3*, *e9292* tomato mutant, created from M82 by chemical mutagenesis using EMS; GFP, green fluorescent protein; YFT3-GFP, a fusion protein: YFT3 protein was fused at the amino terminal of GFP; YFT3 allele-GFP, a fusion protein: YFT3 allele protein was fused at the amino terminal of GFP; CD3-999-mecherry, plasmid abbreviation of pt-rk-CD3-999, which expressed a marker protein, mecherry anchoring on the plastid within plant cells, and in the plasmid of pt-rk-CD3-999, pt, r and k indicate plastid, mCherry, and kanamycin selection, respectively; CD3-999 indicates ABRC stock number (http://www.arabidopsis.org). Anti-YFT3, rabbit anti-YFT3 polyclonal antiserum; Anti-actin, rabbit anti-β-actin (plant) monoclonal antibody. 35 dpa, 47 dpa, and 54 dpa correspond to the mature green stage (MG), breaker stage (BR), and red/yellow ripening stage (RR/YR) during tomato fruit ripening.

To determine the impact of Ser126Arg substitution on the subcellular localization and products of YFT3 allele protein, *YFT3-CDS* and *YFT3 allele-CDS* without the stop codon were fused to the N-terminus of the fluorescent reporter GFP to create *35S::YFT3-GFP* and *35S::YFT3/allele-GFP*. The two plasmids were transformed into *Nicotiana benthamiana* leaves, and confocal microscopy data showed that proteins of both YFT3 and YFT3 allele were localized in the chromoplasts (Fig. 5A). Western blot analysis revealed that there were no significant differences between the abundances of YFT3 and YFT3 allele in the fruits of M82 and *yft3* lines at 35, 47, and 54 dpa, although both β-ACTIN and YFT3/YFT3 allele protein contents were significantly decreased at 54 dpa compared with any of the other time points (Fig. 5B, Supplementary Data Fig. S6). These results suggest that the Ser126Arg substitution did not affect the cellular localization of YFT3/YFT3 allele proteins and their abundance in the WT and mutant lines.

### Color complementation and enzymatic activity

**Figure 6:**
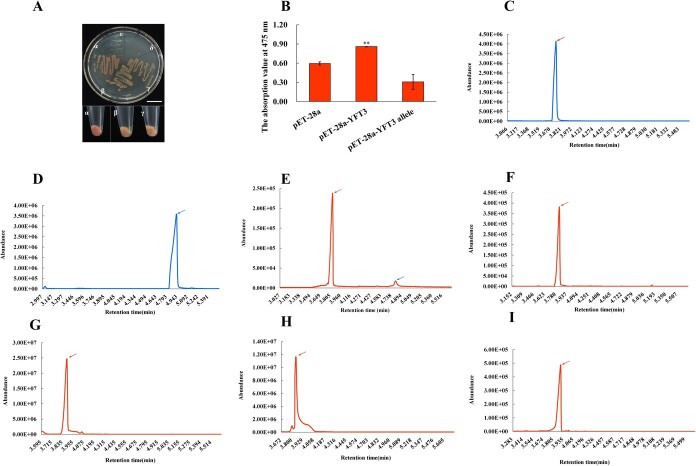
Catalytic activity assay of YFT3 and YFT3 allele *in vivo* and *in vitro.***A** Colors of bacterial plaques and pellets in DE3 (*E. coli*) cells expressing different YFT constructs. α, β, and γ indicate DE3 cells harboring *p*Trc-LYC transformed with the pET28-YFT3 and pET28-YFT3 allele, and pET-28a(+) plasmids, respectively; δ and ε indicate DE3 cells transformed with the empty *pET-28a(+)* and *p*Trc-LYC plasmid, respectively. Scale bar = 2 cm. **B** Lycopene contents in DE3 cells expressing different YFT3 constructs: absorption value at 475 nm of an acetone extract from the IPTG induced DE3 cells harboring pET28-YFT3, pET28-YFT3 allele constructs, and pET-28a(+). **Statistically significant difference determined by Student’s *t*-test at *P* < 0.01. **C** Gas chromatogram of standard 3-methyl-3-butene-1 alcohol (peak 1, 3.8 min). **D** 3-methyl-2-butene-1 alcohol (peak 2, 4.9 min). **E**–**H** Products of reactions catalyzing conversions of 3-methyl-3-butene-1 alcohol into 3-methyl-2-butene-1- alcohol by YFT3, YFT3 allele, denatured YFT3, and denatured YFT3 allele proteins for 3 h *in vitro*. **I** negative control [empty pET-28a(+) vector]. Red and blue arrows indicate retention times at 3.8 min (3-methyl-3-butene-1 alcohol) and 4.9 min (3-methyl-2-butene-1- alcohol).

*Escherichia coli* ED3 cells harboring the plasmid pTrc-LYC, carrying the coding sequences for *crtB*, *crtE*, and *crtL*, can produce lycopene at a basal level [39]. We used this strain to investigate whether *YFT3* or *YFT3 allele* expression can change lycopene production. ED3 cells harboring either pTrc-LYC or pET-28a(+) alone used as negative controls were cultured on/in LB medium supplemented with chloramphenicol and kanamycin. As a result, the growth of these cells was supressed (Fig. 6Aδ and ε). However, three other strains harboring pTrc-LYC/pET-28a(+), pET-28a-YFT3, or pET-28a-YFT3 allele grew well in liquid LB medium and formed bacterial plaques on solid LB medium. The bacterial plaques and pellets exhibited a reddish-brown color when expressing pET-28a-YFT3 in the DE3 strain haboring pTrc-LYC, indicative of lycopene accumulation within the cells, whereas those expressing the pET-28a-YFT3 allele or pET-28a(+) in DE3 strains haboring pTrc-LYC were beige (Fig. 6Aα, β, and γ). The lycopene contents of the latter two strain lines were also significantly lower than that in the strain carrying pTrc-LYC/pET-28a-YFT3 (Fig. 6B). This result suggests that the YFT3 allele has lower enzyme activity than that in WT YFT3 protein.

We next compared the difference in catalytic activities of the recombinant YFT3 allele and YFT3/SlIDI1 proteins *in vitro* in isomerizing IPP into DMAPP. It has been reported that pyrophosphate groups can be readily removed from IPP and DMAPP with alkaline phosphatase, and can be converted into the more stable isoprenol (3-methyl-3-buten-1-ol, alcohol derivative of IPP) and isopentenyl alcohol (3-methyl-2-buten-1-ol, DMAPP alcohol derivative), which can be easily quantified by GC–MS [40]. Our data showed that the GC–MS retention times of 3-methyl-3-buten-1-ol and 3-methyl-2-butene-1-ol standards were 3.8 and 4.9 min, respectively (Fig. 6C and D). When IPP was incubated with recombinant YFT3 protein after alkaline phosphatase addition, we also determined the retention times of isoprenol and isopentenyl alcohol at 3.8 and 4.9 min (Fig. 6 E). However, only one isoprenol peak was detected when IPP was incubated with any one of YFT3 allele protein, denatured recombinant YFT3, YFT3 allele proteins, or the negative control (empty pET-28a(+) vector) (Fig. 6F–I). These results suggest that the recombinant YFT3 protein can isomerize IPP to DMAPP, but the the recombinant YFT3 allele protein has much lower ativity.

### Ser126 is an essential site for catalytic activity of YTF3

**Figure 7:**
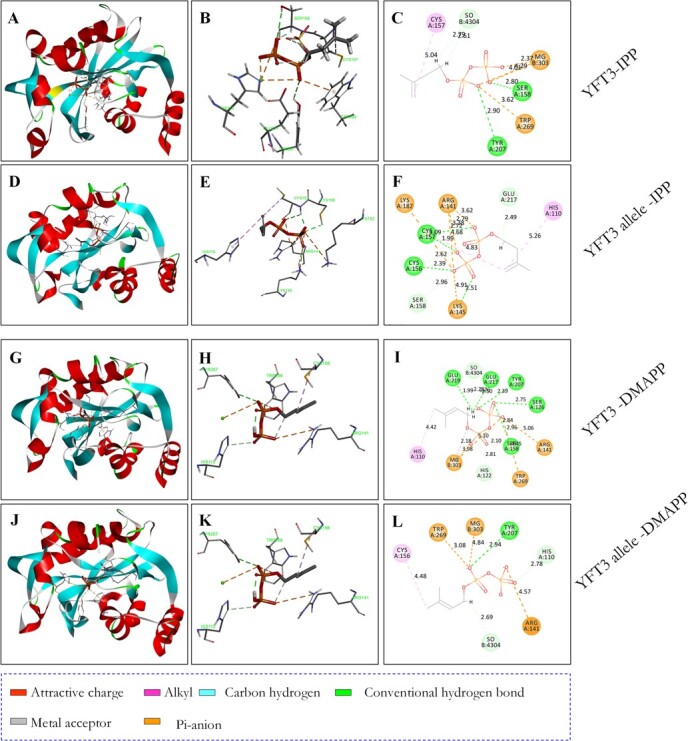
Molecular docking of YFT3 and YFT3 allele proteins with IPP and DMAPP. **A**–**F** Molecular docking of YFT3 and YFT3 allele proteins with IPP. **G**–**L** Molecular docking of YFT3 and YFT3 allele proteins with DMAPP. Regular molecular docking is shown in **A**, **D**, **G**, and **J** and linear molecular docking is shown in **B**, **E**, **H**, and **K**. **C**, **F**, **I**, and **L** are ball-and-stick molecular docking models.

Molecular docking analyses of the YFT3/ YFT3 allele proteins with the IPP/DMAPP substrates was conducted using Discovery Studio 4.5 [41]. It was predicted that the Ser126Arg subsitution alters the conformation of YFT3 and conseqeuntly changes its binding to or interaction with the substrates (Fig. 7). There are four amino acid residues (Cys157, Ser158, Tyr207 and Trp269) in YFT3 conjugating with IPP. Cys157 binds to IPP through the CC double bonds of the alkylate reaction with a bond length of 5.04 Å. Both Ser158 and Tyr207 bind to the IPP pyrophosphate group by conventional hydrogen bonds, with bond lengths of 2.80 and 2.90 Å, respectively. Finally, Trp269 binds to the IPP pyrophosphate group by interacting with the Pi-anion in the polar indole ring, with a bond length of 3.62 Å (Fig. 7A–C, Supplementary Data Table S4). Replacement of Ser126 with Arg in the YFT3 allele protein alters its spatial conformation, and makes YFT3 allele protein bind to IPP through eight amino acid residues (His110, Arg141, Lys145, Cys156, Cys157, Ser158, Lys182, and Glu217) (Fig. 7 D–F). The model indicates that the hydrosulphonyl residues of His110 and Cys157 bind to the CC double bonds of IPP via a Pi-alkyl reaction, and with bond lengths of 5.26 and 4.83 Å. Lys145, Cys156, and Cys157 bind to the pyrophosphate group of IPP via hydrogen bonds, with bond lengths of 2.51, 2.39, 1.99, and 2.72 Å, respectively. Four amino acid residues (Arg141, Cys156, Ser158, and Glu217) conjugate with IPP by hydrogen–carbon bonds, with lengths of 2.79, 2.62, 2.96, and 2.49 Å, respectively. Simultaneously, Arg141, Lys145, and Lys182 also bind to the pyrophosphate group of IPP by attractive charges, and with bond lengths of 3.62, 3.38, and 4.68 Å for Arg141, 4.91 Å for Lys145, and 5.09 Å for Lys182, respectively (Fig. 7D–F, Supplementary Data Table S4).

We also performed a molecular docking analysis of both the YFT3 and YFT3 allele proteins with DMAPP. The Ser126Arg mutation in the YFT3 allele resulted in a decrease in the number of active amino acid residues binding to DMAPP from nine to five. The amino acid residues Tyr207, Glu217, and Glu219 in YFT3 protein bind to the hydrogen atom in DMAPP by conventional hydrogen bonding, with bond lengths of 2.39, 2.50, and 1.99 Å, respectively. YFT3 also conjugates with DMAPP through binding to different oxygen atoms in the pyrophosphoric acid group by six active amino acid residues, both Ser126 and Ser158 with a conventional hydrogen bond, with bond lengths of 2.75 and 2.84 Å; Arg141 and Trp269 via a Pi-anion with bond lengths of 5.06 and 2.96 Å; His122 and Ser158 by a carbon–hydrogen bond with bond lengths of 2.81 and 2.10 Å; and His110 with an alkyl reaction with a bond length of 4.42 Å (Fig. 7G–I, Supplementary Data Table S4). All nine active amino acid residues in the YFT3 protein form a pocket that interacts with DMAPP, located next to the Ser126 active pocket, which is essential for sustaining the optimal conformation between the substrate (DMAPP) and the catalytic enzyme (YFT3) [42].

In contrast, the YFT3 allele protein conjugates with DMAPP by five active amino acids (His110, Arg141, Cys156, Tyr207, and Trp269) (Fig. 7J–L). Specifically, the residues His110, Arg141, Tyr207, and Trp269 were shown to interact with the oxygen atom of the pyrophosphoric acid group in DMAPP via a carbon–hydrogen bond (2.78 Å), an attracting charge with a bond length of 4.57 Å, a hydrogen bond (2.94 Å), and a Pi-anion (3.08 Å), respectively. The residue Cys156 binds to the methylated carbon atom in DMAPP through an alkyl reaction with a bond length of 4.48 Å (Fig. 7J–L, Supplementary Data Table S4).

Mg^2+^ is a key cofactor for YFT3 folding into an active conformation, and YFT3 protein also ensures that two oxygen atoms from the IPP pyrophosphate group bind to Mg^2+^ via attracting charges, with bond lengths of 4.01 and 4.29 Å. However, in addition to the increase in the number of IPP-interacting amino acid residues in the YFT3 allele protein, Ser126Arg replacement is also predicted to disrupt the interaction between IPP and Mg^2+^ (Fig. 7C and F). Mg^2+^ also participates in the interaction between YFT3 and DMAPP though binding to three oxygen atoms of the Pi-anion (two oxygen atoms) and a metal acceptor (one oxygen atom) (Fig. 7I). However, in the case of the YFT3 allele protein, Mg^2+^ was predicted to bind to an oxygen atom in the pyrophosphoric acid group in DMAPP via only the Pi-anion, with a bond length of 4.84 Å (Fig. 7L). These results suggest that Ser126Arg replacement increases the number of active amino acid residues of the YFT3 allele protein interacting with IPP, but decreases the number of active amino acid residues with DMAPP. Similarly, the Ser126Arg mutation also results in the disruption of the interaction between IPP and Mg^2+^ during the YFT3 allele isomerization process, and affects binding of DMAPP to Mg^2+^ as well.

## Discussion

Fruit color is one of the most important quality traits, and in tomatoes it is closely associated with carotenoid accumulation [6, 10, 43], particularly the ratio of lycopene to β-carotene [11]. Yellow- and orange-fruited tomatoes are usually deficient in carotenoids, especially lycopene accumulation, which gives rise to the red color. In this study we characterized the genetic molecular basis of a yellow-fruited tomato mutant, *yft3*/*e9292*. A *YFT3 allele* gene with a genetic lesion was identified in the *yft3* mutant using map-based cloning, and a missense mutation (A2117C) downstream of the start codon was found. This point mutation results in a Ser126Arg substitution in the YFT3 allele protein (Fig. 1B). The *YFT3* locus was mapped to chromosome 4, and it was predicted to be the candidate *SlIDI1* gene, encoding an IDI1 enzyme that catalyzes isomerism between IPP and DMAPP in the MEP pathway. *IDI1* derived from tobacco was initially isolated by Nakamura *et al*. [44]. *SlIDI1* was firstly cloned from tomato by Pankratov *et al*. [4], and three mutation forms were identified, which resulted in reduction of carotenoid accumulation. The three mutants *fruit carotenoid deficient 1* (*fcd1*)-*1*, *fcd1-2*, and *fcd1-3* comprise an eliminated W206Δ tryptophan in *fcd1-1*(*e1535*), a W143* nonsense mutation in *fcd1-2* (*e0321* and *e9292*), and a G207R missense mutation in *fcd1-3* (*e0955*). Zhou *et al*. [2] reported that the TB735 tomato (*S. lycopersicum*) also exhibited a significant reduction in carotenoid content due to a 116-bp deletion in *oft3* (*IDI1 allele*). However, in the current study the lycopene content (2.24 μg g^−1^ FW) in the *yft3* mutant was only 2.5% of that in WT M82 tomato. It is notable that the lycopene content in *yft3* is far less than that in *fcd1-2* [4] or *oft3* [2]. Both the *fcd1-2* and *oft3* tomato lines exhibited premature translation termination of SlIDI1 proteins, but unlike these mutations that cause protein truncation, our *yft3* mutation is a substitution of Ser126 Arg, which is an essential amino acid residue for YFT3 function.

As a member of the Nudix hydrolase family, IDI (E.C. 5.3.3.2) is a rate-limiting enzyme that catalyzes a crucial activation step in the isoprenoid biosynthetic pathway responsible for sterol, carotenoid, dolichol, ubiquinone, and prominent classes of prenylated protein synthesis [45]. Activity-enhancing mutations in an IDI triple mutant (L141H/Y195F/W256C) from *Saccharomyces cerevisiae* were identified by error-prone PCR [39]. Three amino acid residues, Cys-67, Tyr-104, and Glu-116 in ElIDI1 from *E. coli*, had been revealed to be involved in the interaction between the divalent metal (Mg^2+^ and Mn^2+^) and the IPP and DMAPP substrates via protonation/deprotonation [46]. The four amino acid residues (Cys87, Glu149, Trp197, and Tyr137) in *Homo sapiens* HsIDI were confirmed to be indispensable for the stereo-selective antarafacial transposition of a proton to convert IPP to DMAPP [45]. However, only a few active amino acid residues have been identified in tomatoes, although *fcd1-1* (Gly216Arg) and *fcd1-3* (Trp215Δ) exhibited a slight decrease in carotenoid accumulation [4]. YFT3/SlIDI1 was found here to be specifically localized in the plastids, as was the YFT3 allele protein (Fig. 5A). The YFT3 allele protein was also produced in the *yft3* mutant at similar abundance to WT YFT3 protein in M82 (Fig. 5B).

However, the expression of the *YFT3 allele* gene was significantly higher in *yft3* fruit than in *YFT3* in M82 during tomato fruit ripening (Fig. 2B), as was the expression of eight genes associated with MEP (*DXR*, *DXS*, and *HDR*) and CSP (*PSY1*, *CRTISO*, *CYCB*, *CYP97A*, and *NCED*), but excluding *HDR* and *CYP97A* at 54 dpa (Fig. 4). In the *YFT3-KO* lines, the transcript levels of the genes associated with MEP (*DXR*, *DXS*, and *HDR*) and CSP (*PSY1*, *CYCB*, *CRITSO*, *CYP97A*, and *NCED*) were also significantly higher than that in M82 at the same time points, except for *HDR*, *PSY1*, and *CRTISO* at 54 dpa (Fig. 4). These results suggest that some genes associated with MEP and CSP were expressed in yellow-fruited tomato lines, such as *yft3* and *YFT3-KO-10/14*, which is counterintuitive based on the low carotenoid levels in *yft3* and *YFT3-KO* lines*.* However, we did not detect significant differences among the red-fruit tomato lines in the transcript levels of most of the eight genes associated with carotenoid synthesis, whereas transcript levels of *CYP97A* in both *35S::YFT3-CP-6/16* and *35S::YFT3-OE-3/6/7* lines at 47 dpa and *DXR*, *DXS*, and *PSY1* at 54 dpa were significantly higher than that in M82 at the same time points, but expressions of *HDR* and *CYP97A* in the *35S::YFT3-CP-6/16* and *35S::YFT3-OE-3/6/7* lines were significantly lower than that in M82 at 54 dpa (Fig. 4).

Fruit color formation is an intricate biological process in tomato, but its molecular regulation remained largely unknown. We hypothesize that there may be a compensation mechanism for *YFT3* with genetic lesions resulting in increased expression of some genes associated with carotenoid synthesis in *yft3* and *YFT3-KO* lines, potentially by feedback regulation from the chromoplasts of the yellow-fruited tomato lines.

As five-carbon basic building units, isopentenyl diphosphate (IPP) and its isomer dimethylallyl diphosphate (DMAPP) are common isomeric precursors for all isoprenoids/terpenoids [33]. One active DMAPP molecule was respectively condensed with one IPP, two IPPs, and three IPPs to form C10 geranyl diphosphate (GPP), C15 farnesyl diphosphate (FPP), and C20 geranyl diphosphate synthase (GGPP), which is an essential precursor to produce various terpenoids/isoprenoids such as C40 carotenoids [47]. Therefore, a huge sink would be formed to store various secondary metabolites such as carotenoids with tomato fruit ripening. However, IPP and DMAPP were produced at a ratio of 6:1 in the MEP pathway in plastids by HMBPP reductase (HDR) catalysis [4]. The ratio or balance of IPP to DMAPP would be disturbed with isoprenoid synthesis, and will result in deficiency of DMAPP, especially if SlIDI1 protein function, which catalyzes conversion between IPP and DMAPP, is lost or its activity decline, as in like *yft3* and *YFT3-KO* line. To meet the powerful traction from the huge sink and isoprenoid syntheses, more DMAPP would need to be produced in plastids, and thus the genes associated with MEP would be triggered or promoted to express it. In particular, the YFT3 protein function presents decline or loss in *yft3* and *YFT3-KO* tomato lines, which will block or abolish conversion from IPP into DMAPP, and this will result in a decline in DMAPP content within plastids. More DMAPP would need to be produced for isoprenoid syntheses, and this requirement will enhance transcript expression of some genes associated with MEP and CSP in *yft3* and *YFT3-KO* lines by a feedback regulation pattern (Fig. 4).

We used an engineered *E. coli* strain to express genes related to carotenoid synthesis to assess the catalytic activity of YFT3 and its allele protein, with the latter showing reduced activity (Fig. 6). Our GC–MS analysis further showed that the recombinant YFT3 protein could convert IPP to DMAPP *in vitro*, while the recombinant YFT3 allele and denatured YFT3/YFT3 allele proteins failed to do this (Fig. 6). These results indicate that the Ser126Arg missense mutation abolishes the ability of YFT3 allele to convert IPP to DMAPP. To further elucidate the basis and functional lesion of the YFT3 allele protein, we performed a molecular docking analysis of its interaction with the IPP/DMAPP substrates. The results suggest that the Ser126Arg mutation resulted in changes in the spatial conformation of the enzyme–ligand interaction in the YFT3 allele protein, with the number of amino acid residues binding to IPP increasing from four to eight, while binding to DMAPP decreased from nine to five. The analysis also predicted that the mutated YFT3 allele protein disrupts IPP binding to the Mg^2+^ cofactor. Those results would explain the lower catalytic activity compared with that of YFT3 protein (Fig. 7).

Based on our results, we predicted that Ser126 is an essential amino acid residue for the function of YFT3/SlIDI1, which is a rate-limiting enzyme in the isoprenoid biosynthetic pathway and catalyzes the reversible conversion between IPP and DMAPP in the plastids. Thereby, Ser126Arg in YFT3 allele would affect carotenoid accumulation in tomato fruit. This study provides important insights into tomato quality improvement and breeding in the future.

## Materials and methods

### Plant materials and growth conditions

Seeds of both WT (*S. lycopersicum*, cv. M82) and *e9292* (*S. lycopersicum*, named *yellow fruited tomato 3*, *yft3*) mutant were provided by Professor Dani Zamir (the Hebrew University of Jerusalem). The *yft3* mutant was created from M82 by mutagenesis with ethyl methyl sulfonate (EMS). Seeds of LA1585 (*Solanum pimpinellifolium*) were obtained from the Tomato Genetics Resource Center (University of California, Davis, CA, USA). Tomato seeds of two mutant populations (*yft3* × LA1585 and M82 × *yft3*) from different generations (*F*_1_ and *F*_2_) were created by sexual hybridization. Transgenic tomato lines of *YFT3-KO*, *35S::YFT3-OE*, and *35S::YFT3-CP* were generated in the M82 and *yft3* background by *Agrobacterium tumefaciens*-mediated transformation. All tomato lines were planted and grown under standard greenhouse conditions at the Pujiang experimental base (121°30′10.89″ E, 31°3′5.20″ N, altitude 5 m), at the Shanghai Jiao Tong University, Shanghai, China. Tobacco (*Nicotiana benthamiana*) seeds were stored in Zhao Lab at the Shanghai Jiao Tong University, China. Tobacco seedlings were grown in pots with damp nutrient soil (field soil:vermiculite:humus = 4:2:4) in a chamber at 24°C under a 16-h light/8-h dark light regime with 20 000 lux and 65% relative humidity.

### Analyses of genetics of fruit colors and gene map-based cloning

Based on the external ripening fruit colors (red and yellow) of tomato plants in the *F*_1_ and *F*_2_ generations (*yft3* × LA1585 and M82 × *yft3*), the inheritance of fruit color in the *yft3* mutant was analyzed using a χ^2^ test. To make linkage groups with the mutated loci, a total of 45 CAPS/dCAPS markers were created based on data from the Sol Genomics Network database (http://solgenomics.net/). These markers span all 12 tomato chromosomes, and were designed by analyzing single-nucleotide polymorphisms (SNPs) in the target DNA sequences between *yft3* and LA1585 (Supplementary Data Table S1). The *yft3* × LA1585 tomato plants with different fruit colors in the segregating *F*_2_ generation were also used to identify the candidate gene by map-based cloning. Genomic DNA was extracted from the young leaves of each plant as described in Chen *et al*. [11]. Forty-five markers were used to screen 116 individual plants randomly selected from *yft3* × LA1585 *F*_2_ population (25 yellow-fruited and 91 red-fruited), for primary mapping of the location of the *YFT3* gene. Based on the genotypes and fruit color phenotypes of 116 plants in the *F*_2_ generation, the target region was confirmed by calculating the LOD scores. An additional 1338 plants derived from the *yft3* × LA1585 *F*_2_ population were used to fine-map the *YFT3* target region using seven newly-designed CAPS markers (Supplementary Data Table S1).

Genotypic and phenotypic data from the *F*_2_ population were used to create linkage maps using R/QTL software [48], and the region with the candidate *YFT3* gene was identified using the Genome Browser (https://solgenomics.net/jbrowse_solgenomics). The candidate gene was further confirmed by examining the gene functional annotations in the predicted mapping region https://solgenomics.net/jbrowse_solgenomics). A DNA fragment that contains the candidate gene, *ISOPENTENYL DIPHOSPHATE ISOMERASE 1* (*IDI1*), was amplified using LA Taq DNA polymerase (Takara, Dalian, China) with the gene-specific primers 5′-cacccttaggttggtgttttgttgag-3′ (forward) and 5′-gcctaatctgaaatggctcaaagg-3′ (reverse) (Supplementary Data Table S3) and sequenced.

### Structural features of *YFT3*

Total RNA was extracted from fresh pericarp at the equatorial region of M82 and *yft3* tomato fruits at 47 dpa (corresponding to the breaker stage, BR), using the RNAprep Pure Plant Kit (Tiangen, Beijing, China). The coding sequences (CDS) of *YFT3* and *YFT3 allele* were amplified from M82 and *yft3* tomato fruit cDNA libraries (at 47 dpa) using specific primer pairs (Supplementary Data Table S3). The CDS sequences were aligned to the corresponding genomic DNA sequence to confirm the number and length of exons and introns, as well as the mutations in *YFT3 allele* in *yft3* and the corresponding amino acid substitutions.

### Constructs and genetic transformation of tomatoes

The *YFT3* CDS was amplified from a red-fruited M82 cDNA library (at 47 dpa) using the specific primers 35S-CDS-BamHI and 35S-CDS-SacI (Supplementary Data Table S3), and then cloned into the BamHI and SacI sites in an intermediate vector plasmid *35S::GUS* to create *35S::YFT3-CDS.* The *35S::GUS* expression vector was constructed based on the backbone of the pCAMBIA 2300 plasmid, and with the *GUS* expression cassette from pBI121 being inserted into the corresponding HindIII and EcoRI sites of the pCAMBIA 2300 plasmid.

The *YFT3-KO* vector was constructed based on the pTX041 plasmid [49], provided by Professor Chuanyou Li (Institute of Genetics and Developmental Biology, Chinese Academy of Sciences, Peking, China). Two target DNA fragments for the *YFT3-CDS* sequence were designed using the CRISPR-P v2.0 website (http://cbi.hzau.edu.cn/CRISPR2/). Specific *cri-YFT3*-F/R primers (Supplementary Data Table S3) were designed to amplify the DNA fragments with two target sequences with pTX043 as the template using Ex Taq DNA Polymerase (TaKaRa, Dalian, China). The amplified PCR product was digested with BsaI and then constructed into the corresponding pTX041 site to create *YFT3-KO*.

The *35S::YFT3-CDS* and *YFT3-KO* plasmids were separately introduced into *A. tumefaciens* (strain EHA105) using the freeze–thaw method [50], and then used to transform *yft3* or M82 tomato lines as described [51]. Three transgenic tomato lines were respectively designated *35S::YFT3-CP* (functional complementation of the *YFT3 allele* in *yft3* tomato), *35S::YFT3-OE* (*YFT3* overexpression in M82), and *YFT3-KO* (*YFT3* knockout in M82). Fruit colors of the transgenic tomato lines were examined at 35, 47, and 54 dpa.

### Subcellular localization of YFT3 protein

*YFT3-CDS* and *YFT3 allele-CDS* were respectively amplified from cDNA libraries of M82 and *yft3* tomato fruit (47 dpa) with the specific primers *YFT3-GFP-*BamHI/*YFT3-GFP-*SpeI (Supplementary Data Table S3). The validated PCR fragments were inserted into the same sites in the pHB plasmid [52] to generate *2 × 35S::YFT3-CDS-GFP* and *2 × 35S::YFT3 allele-CDS-GFP* constructs.

The *2 × 35S::YFT3-CDS-GFP*, *2 × 35S::YFT3 allele-CDS-GFP*, *2 × 35S::pt-rk-CD3–999* [53], and *35S::p19* [54] plasmids were individually introduced into *A. tumefaciens* (strain GV3101) using the freeze–thaw method [50]. Bacterial cultures were grown in a shaker at 250 rpm and 28°C until they reached an OD_600_ value of 0.6–0.8. Cells were collected by centrifugation at 4000 × *g* for 10 min and resuspended in 10 mM MgCl_2_ to an OD_600_ of 0.6, and 2-*N*-morpholino ethanesulfonic acid (MES) and acetosyringone (AS) were added to a final concentration of 10 mM and 40 μM, respectively, and the cell suspensions were shaken at 250 rpm in the dark for 3 h.

Bacteria harboring *2 × 35S::YFT3-CDS-GFP* or *2 × 35S::YFT3 allele-CDS-GFP* were mixed with *2 × 35S::pt-rk-CD3–999* [53] and *35S::p19* in a ratio of 1:1:2 (v:v:v). Tobacco (*N. benthamiana*) leaves were transformed as described [51] and then placed in a dark condition at 25°C for 24 h before being transferred to light for another 24 h. The transformed leaves were sampled to observe the GFP and mCherry signals under a fluorescence confocal microscope (FCM, TCS SP5, Leica, Germany). The excitation and emission wavelengths of 488 and 507 nm, respectively, were selected for GFP, and 543 and 568 nm for mCherry.

### Visualization of chromoplast ultrastructure

Fruits were collected from *YFT3-CDS-CP*, *YFT3-CDS-OE*,*YFT3-KO*, M82, and *yft3* tomato lines (35, 47, and 54 dpa, *n* = 3), and the pericarps of the equatorial region were sampled. The preparation, treatment, and examination of samples for chromoplast ultrastructure were conducted as described in Zhao *et al*. [29].

### Real time–quantitative PCR analysis

Total RNA samples were extracted from roots, stems, leaves, sepals, petals, anthers, pistils, and fruit (35, 47, and 54 dpa) of M82 and *yft3* (*n* = 3), as well as fruit (35, 47, and 54 dpa) of *YFT3-CDS-CP*, *YFT3-CDS-OE*, and *YFT3-KO* tomato lines (*n* = 3), using an RNAprep Pure Plant Kit (Tiangen, China). The total RNA samples were treated with RNA-free DNase I (New England BioLabs, http://www.neb.com) to remove trace genomic DNA. The RT–qPCR analysis was performed as described in Zhao *et al*. [29]. The gene expression levels were calculated using the 2^−ΔCT^ equation with *ACTIN* (*Solyc03g078400*) as an internal reference gene for normalization [55]. The transcript expression levels of genes involved in the MEP pathway (*DXR*, *DXS*, *HDR*, and *SlIDI1*) and CSP pathway (*PSY1*, *CRTISO*, *CYCB*, *CPY707A*, and *NCED*) were determined in the current study, and all RT–qPCR primers are listed in Supplementary Data Table S3.

### Measurement of carotenoid content

Pericarp samples from the equatorial region of the *YFT3-CDS-CP/OE*, *YFT3-KO*, M82, and *yft3* fruits were collected at 35, 47, and 54 dpa, and then powdered in liquid nitrogen. Carotenoids were extracted using methyl alcohol/chloroform, and examined using a Waters Acquity Ultra-performance Convergence Chromatography (UPC^2^) system (Waters, Milford, MA, USA) as described in Zhao *et al*. [29]. The standards of lycopene, β-carotene, α-carotene, and lutein were products of Yuanye Biotechnology (Shanghai, China). The standards were dissolved in MTBE to make standard curves, which were drawn as described in Zhao *et al*. [29].

### Extraction of total soluble protein and western blotting

Pericarp samples of the equatorial region of M82 and *yft3* tomatoes (35, 47, and 54 dpa) were collected (*n* = 3) and powdered in liquid nitrogen. Approximately 1 g of pericarp powder was mixed well with 1 ml PBS extraction buffer (1.75 mM KH_2_PO_4_, 10 mM Na_2_HPO_4_, 140 mM NaCl, 2.7 mM KCl, pH 7.4) with 1 mM phenylmethylsulfonyl fluoride (PMSF, Yeasen, Shanghai, China) to extract total soluble protein (TSP). The homogenates were placed in an ice bath for 4 h and centrifuged at 12 000 × *g* at 4°C for 40 min. The supernatants were collected, and the TSP concentrations were measured using the Bradford method [56]. Bovine serum albumin was used to create a standard curve.

The concentration of crude TSP was adjusted to 0.5 μg/μl with PBS extraction buffer, and 10 μl of TSP was mixed with 5× loading buffer [250 mM Tris–HCl (pH 6.8), 50% (v/v) glycerol, 10% (w/v) sodium dodecyl sulphate (SDS), 5% (v/v) β-mercaptoethanol, and 0.5% (w/v) bromophenol blue] and was denatured at 95°C for 10 min before centrifugation at 10 000 × *g* for 1 min. All protein samples were separated using 10% SDS/polyacrylamide gel electrophoresis (SDS–PAGE) in Tris–glycine buffer [0.025 M Tris, 0.25 M glycine and 0.01% (w/v) SDS]. Subsequently, one gel was stained with 2.5% (w/v) Coomassie Brilliant Blue R250, and the other gel loaded with protein samples in the same order was transferred to a polyvinylidene fluoride (PVDF) membrane (filter pore size 0.22 μm, Millipore, USA) for western blot analysis. The PVDF membrane was blocked in 5% (w/v) non-fat milk powder (TPBS, Sangon Biotech, Shanghai, China) for 2 h. The YFT3 protein was detected by incubating the transferred PVDF membrane with a rabbit anti-YFT3 polyclonal antiserum, which was created by using an oligomeric peptide of RGIDGNKPMSLTTAS located at amino acids 2–16 of the YFT3 protein, to immunize rabbits (Sangon Biotech, Shanghai, China) at a 1:500 dilution in TPBS at 4°C overnight. Horseradish peroxidase (HRP)-conjugated goat anti-rabbit immunoglobulin G (IgG) (Beyotime, Shanghai, China) at a 1:1000 dilution in TPBS was used as the secondary antibody. Immunoreactive YFT3 protein was visualized using the Ultra High Sensitivity ECL Kit (MedChemExpress, Shanghai, China) and scanned to produce digital images. Tomato β-actin (http://www.affbiotech.cn/) was used as a reference protein.

### *In vivo* and *in vitro* enzymatic activity of YFT3

The *pTrc-LYC* plasmid containing a chloramphenicol resistance gene was constructed from the *pTrcHis2B* framework plasmid, which carries a gene cluster of *crtE* (geranylgeranyl pyrophosphate synthase), *crtB* (phytoene synthase), and *crtL* (lycopene cyclase) for lycopene biosynthesis [39]. It was kindly provided by Professor Haibo Zhang (Qingdao Institute of Bioenergy and Bioprocess Technology, Chinese Academy of Sciences, Qingdao, China). *pTrc-LYC* derived from a prokaryotic expression system was used to estimate enzymatic activity of the YFT3 protein [57].

The amplified *YFT3* and *YFT3 allele* CDSs from M82 and *yft3* tomato fruit cDNA libraries were individually constructed at BamHI and SacI sites in the *pET-28a*(+) plasmid (Yeasen Biotechnology, Shanghai, China) using an InFusion kit (Vazyme Biotechnology, Nanjing, China), to create plasmid vectors of *pET-28a-YFT3* and *pET-28a-YFT3 allele.* The *pET-28a-YFT3*, *pET-28a-YFT3 allele*, and *pET-28a*(+) plasmids were then introduced into Rosetta (DE3, *E. coli*) cells (Weidi Biotechnology, Shanghai, China) carrying *pTrc-LYC*. DE3 cells harboring either *pET-28a*(+) or *pTrc-LYC* alone were used as negative controls.

The DE3 cells were cultured in lysogeny broth (LB) liquid medium [10 g/l tryptone and 5 g/l yeast extract (Thermo Fisher, USA), and 10 g/l NaCl (Lingfeng，Shanghai, China), pH 7.0] with 50 mg/l chloramphenicol and kanamycin, and grown with shaking at 250 rpm at 37°C until cultures reached an OD_600_ value of 1.0. Isopropyl-beta-d-thiogalactopyranoside (IPTG) was added to a final concentration of 0.5 mM, and then the cells were spread on a Petri dish with LB/agar containing 100 mg/l chloramphenicol and kanamycin. The plates were placed upside down in an incubator (Tiancheng, Shanghai, China) in the dark at 28°C for 3 days. The proliferation and color of the cells were observed each day.

Each DE3 engineered strain was inoculated in 100 ml LB liquid medium with 50 mg/l chloramphenicol and kanamycin, and shaken at 220 rpm at 37°C to an OD_600_ of 0.6.

IPTG was then added to a final concentration of 0.5 mM and the cultures were shaken at 100 rpm at 16°C to induce recombinant protein expression. Five milliliters of each culture was sampled 24 h after induction, and the cells were collected by centrifugation at 3000 × *g* for 5 min. The cell pellet was resuspended in 200 μl acetone and incubated in a water bath at 55°C for 15 min, and centrifuged at 10 000 × *g* for 3 min; 150 μl of the supernatant was sampled and absorbance was measured at 475 nm (PowerWave XS, Biotek, VT, USA). The relative lycopene ratio (RLR) was calculated using the formula: RLR = (*Y*_TRIAL_ − *Y*_CONTROL_)/*Y*_CONTROL_. Here, *Y*_TRIAL_ is the absorbance value for DE3 cells concurrently harboring *pTrc-LYC*, *pET-28a-YFT3* or *pET-28a-YFT3* allele, and *Y*_CONTROL_ is the absorbance value for the DE3 cells carrying both *pTrc-LYC* and *pET-28a*(*+*).

The activities of the recombinant YFT3 and YFT3 allele proteins were estimated *in vitro*, as described in Ma *et al*. [40] with some modifications. The purified recombinant YFT3 and YFT3 allele proteins and 10 μg of the empty pET-28a(+) vector were individually added to a 1.5-ml Eppendorf tube containing 5 mM MgCl_2_, 1 mM DTT, and 20.0 μg IPP substrate. The volume was adjusted to 100 μl by adding 50 mM PBS. The reaction was performed at 37°C in darkness for 3 h, and then 1 U alkaline phosphatase (Shanghai Yuanye Biotechnology, Shanghai, China) was added. The reactions were incubated at 37°C overnight to completely remove the pyrophosphate group from IPP or DMAPP, and the reaction products were extracted by addition of petroleum ether (v:v = 1:1).

The enol derivatives of IPP and DMAPP, 3-methyl-3-butene-1-ol and 3-methyl-2-butene-1-ol are more stable than IPP and DMAPP. The concentrations of these derivatives were assayed by gas chromatography–mass spectrometry (GC–MS, Agilent, CA, USA) to estimate enzymatic catalytic activity. GC–MS analysis was performed using a low-loss HP-5-ms (Agilent, California, USA) GC–MS column. The initial gas chromatography temperature was 35°C for 2 min, and then increased to 40°C at 5°C/min before holding for 5 min. Finally, 280°C was reached at 20°C/min and held for 5 min. The mass spectral scan range was set to 30–240 *m*/*z*.

### Molecular docking analysis

To elucidate structural differences between the YFT3 and YFT3 allele proteins that might explain their different catalytic activities, we predicted the binding pockets of the two proteins and the substrates IPP/DMAPP using Discovery Studio 4.5 (DS) [41]. A homology model of YFT3 was created using human isopentenyl diphosphate isomerase (hIDI) (PDB code: 2ICJ) as a reference template. The target protein was minimized by invoking the functions of Minimize and Refine in DS [58]. IPP and DMAPP structures were drawn using ChemDraw. The CDOCKER module available with DS was employed to generate the interaction model between the YFT3/YFT3 allele catalytic proteins and IPP/DMAPP substrates with all the parameters set as default [59]. The best position was selected based on the highest docking score from the largest cluster and the key residue interactions [60].

## Supplementary Material

Web_Material_uhae202

## References

[1] Liu LH , Shao ZY, Zhang M. et al. Regulation of carotenoid metabolism in tomato. *Mol Plant*. 2015;8:28–39 25578270 10.1016/j.molp.2014.11.006

[2] Zhou M , Deng L, Guo SG. et al. Alternative transcription and feedback regulation suggest that SlIDI1 is involved in tomato carotenoid synthesis in a complex way. *Hortic Res*. 2022;9:uhab045 35031800 10.1093/hr/uhab045 PMC8788357

[3] Botella-Pavía P , Besumbes O, Phillips MA. et al. Regulation of carotenoid biosynthesis in plants: evidence for a key role of hydroxymethylbutenyl diphosphate reductase in controlling the supply of plastidial isoprenoid precursors. *Plant J*. 2004;40:188–99 15447646 10.1111/j.1365-313X.2004.02198.x

[4] Pankratov I , McQuinn R, Schwartz J. et al. Fruit carotenoid-deficient mutants in tomato reveal a function of the plastidial isopentenyl diphosphate isomerase (IDI1) in carotenoid biosynthesis. *Plant J*. 2016;88:82–94 27288653 10.1111/tpj.13232

[5] Zhong SL , Fei ZJ, Chen YR. et al. Single-base resolution methylomes of tomato fruit development reveal epigenome modifications associated with ripening. *Nat Biotechnol*. 2013;31:154–9 23354102 10.1038/nbt.2462

[6] Sun TH , Rao S, Zhou XS. et al. Plant carotenoids: recent advances and future perspectives. *Mol Hortic*. 2022;2:3 37789426 10.1186/s43897-022-00023-2 PMC10515021

[7] Schwartz SH , Qin XQ, Zeevaart JAD. Characterization of a novel carotenoid cleavage dioxygenase from plants. *J Biol Chem*. 2001;276:25208–11 11316814 10.1074/jbc.M102146200

[8] Vogel JT , Tan BC, McCarty DR. et al. The carotenoid cleavage dioxygenase 1 enzyme has broad substrate specificity, cleaving multiple carotenoids at two different bond positions. *J Biol Chem*. 2008;283:11364–73 18285342 10.1074/jbc.M710106200

[9] Vogel JT , Tieman DM, Sims CA. et al. Carotenoid content impacts flavor acceptability in tomato (*Solanum lycopersicum*). *J Sci Food Agric*. 2010;90:2233–40 20661902 10.1002/jsfa.4076

[10] Tieman D , Zhu GT, Resende MFR Jr. et al. A chemical genetic roadmap to improved tomato flavor. *Science*. 2017;355:391–4 28126817 10.1126/science.aal1556

[11] Chen LL , Li WZ, Li YP. et al. Identified trans-splicing of *YELLOW-FRUITED TOMATO 2* encoding the PHYTOENE SYNTHASE 1 protein alters fruit color by map-based cloning, functional complementation and RACE. *Plant Mol Biol*. 2019;100:647–58 31154655 10.1007/s11103-019-00886-y

[12] Fray RG , Grierson D. Identification and genetic analysis of normal and mutant phytoene synthase genes of tomato by sequencing, complementation and co-suppression. *Plant Mol Biol*. 1993;22:589–602 8343597 10.1007/BF00047400

[13] Ronen G , Cohen M, Zamir D. et al. Regulation of carotenoid biosynthesis during tomato fruit development: expression of the gene for lycopene epsilon-cyclase is down-regulated during ripening and is elevated in the mutant *Delta*. *Plant J*. 1999;17:341–51 10205893 10.1046/j.1365-313x.1999.00381.x

[14] Ronen G , Carmel-Goren L, Zamir D. et al. An alternative pathway to β-carotene formation in plant chromoplasts discovered by map-based cloning of *Beta* and *old-gold* color mutations in tomato. *Proc Natl Acad Sci USA*. 2000;97:11102–7 10995464 10.1073/pnas.190177497 PMC27155

[15] Isaacson T , Ronen G, Zamir D. et al. Cloning of *tangerine* from tomato reveals a carotenoid isomerase essential for the production of beta-carotene and xanthophylls in plants. *Plant Cell*. 2002;14:333–42 11884678 10.1105/tpc.010303 PMC152916

[16] Barry CS , Giovannoni JJ. Ethylene and fruit ripening. *J Plant Growth Regul*. 2007;26:143–59

[17] Gao L , Zhao WH, Qu HO. et al. The *yellow-fruited tomato 1* (*yft1*) mutant has altered fruit carotenoid accumulation and reduced ethylene production as a result of a genetic lesion in *ETHYLENE INSENSITIVE2*. *Theor Appl Genet*. 2016;129:717–28 26743523 10.1007/s00122-015-2660-4

[18] Wang WH , Wang YY, Chen T. et al. Current insights into post-transcriptional regulation of fleshy fruit ripening. *Plant Physiol*. 2023;192:1785–98 36250906 10.1093/plphys/kiac483 PMC10315313

[19] Martel C , Vrebalov J, Tafelmeyer P. et al. The tomato MADS-box transcription factor RIPENING INHIBITOR interacts with promoters involved in numerous ripening processes in a COLORLESS NONRIPENING-dependent manner. *Plant Physiol*. 2011;157:1568–79 21941001 10.1104/pp.111.181107 PMC3252172

[20] Vrebalov J , Ruezinsky D, Padmanabhan V. et al. A *MADS-box* gene necessary for fruit ripening at the TOMATO RIPENING-INHIBITOR (Rin) locus. *Science*. 2002;296:343–6 11951045 10.1126/science.1068181

[21] Manning K , Tör M, Poole M. et al. A naturally occurring epigenetic mutation in a gene encoding an SBP-box transcription factor inhibits tomato fruit ripening. *Nat Genet*. 2006;38:948–52 16832354 10.1038/ng1841

[22] Gao Y , Wei W, Fan ZQ. et al. Re-evaluation of the *nor* mutation and the role of the NAC-NOR transcription factor in tomato fruit ripening. *J Exp Bot*. 2020;71:3560–74 32338291 10.1093/jxb/eraa131 PMC7307841

[23] Vrebalov J , Pan IL, Arroyo AJM. et al. Fleshy fruit expansion and ripening are regulated by the tomato SHATTERPROOF gene *TAGL1*. *Plant Cell*. 2009;21:3041–62 19880793 10.1105/tpc.109.066936 PMC2782289

[24] Fujisawa M , Nakano T, Shima Y. et al. A large-scale identification of direct targets of the tomato MADS box transcription factor RIPENING INHIBITOR reveals the regulation of fruit ripening. *Plant Cell*. 2013;25:371–86 23386264 10.1105/tpc.112.108118 PMC3608766

[25] Fujisawa M , Shima Y, Higuchi N. et al. Direct targets of the tomato-ripening regulator RIN identified by transcriptome and chromatin immunoprecipitation analyses. *Planta*. 2012;235:1107–22 22160566 10.1007/s00425-011-1561-2

[26] Fujisawa M , Shima Y, Nakagawa H. et al. Transcriptional regulation of fruit ripening by tomato FRUITFULL homologs and associated MADS box proteins. *Plant Cell*. 2014;26:89–101 24415769 10.1105/tpc.113.119453 PMC3963596

[27] Lin ZF , Hong YG, Yin MG. et al. A tomato HD-zip homeobox protein, LeHB-1, plays an important role in floral organogenesis and ripening. *Plant J*. 2008;55:301–10 18397374 10.1111/j.1365-313X.2008.03505.x PMC2607530

[28] Chung MY , Vrebalov J, Alba R. et al. A tomato (*Solanum lycopersicum*) *APETALA2/ERF* gene, *SlAP2a*, is a negative regulator of fruit ripening. *Plant J*. 2010;64:936–47 21143675 10.1111/j.1365-313X.2010.04384.x

[29] Zhao WH , Li YH, Fan SZ. et al. The tomato WRKY32 transcription factor affects ripe fruit color by regulating YFT1, a core component of ethylene signal transduction. *J Exp Bot*. 2021;72:4269–82 33773493 10.1093/jxb/erab113

[30] Lanahan MB , Yen Hsiao-Ching C, Giovannoni JJ. et al. The never ripe mutation blocks ethylene perception in tomato. *Plant Cell*. 1994;6:521–30 8205003 10.1105/tpc.6.4.521 PMC160455

[31] Jin X , Baysal C, Gao LH. et al. The subcellular localization of two isopentenyl diphosphate isomerases in rice suggests a role for the endoplasmic reticulum in isoprenoid biosynthesis. *Plant Cell Rep*. 2020;39:119–33 31679061 10.1007/s00299-019-02479-x

[32] Vranová E , Coman D, Gruissem W. Network analysis of the MVA and MEP pathways for isoprenoid synthesis. *Annu Rev Plant Biol*. 2013;64:665–700 23451776 10.1146/annurev-arplant-050312-120116

[33] Athanasakoglou A , Grypioti E, Michailidou S. et al. Isoprenoid biosynthesis in the diatom *Haslea ostrearia*. *New Phytol*. 2019;222:230–43 30394540 10.1111/nph.15586

[34] Ganjewala D , Kumar S, Luthra R. An account of cloned genes of methyl-erythritol-4- phosphate pathway of isoprenoid biosynthesis in plants. *Curr Issues Mol Biol*. 2008;11:i35–45 19193963

[35] Rodriguez-Concepcion M , Boronat A. Elucidation of the methylerythritol phosphate pathway for isoprenoid biosynthesis in bacteria and plastids. A metabolic milestone achieved through genomics. *Plant Physiol*. 2002;130:1079–89 12427975 10.1104/pp.007138 PMC1540259

[36] Rohdich F , Hecht S, Gärtner K. et al. Studies on the non-mevalonate terpene biosynthetic pathway: metabolic role of IspH (LytB) protein. *Proc Natl Acad Sci USA*. 2002;99:1158–63 11818558 10.1073/pnas.032658999 PMC122160

[37] Tritsch D , Hemmerlin A, Bach TJ. et al. Plant isoprenoid biosynthesis via the MEP pathway: in vivo IPP/DMAPP ratio produced by (*E*)-4-hydroxy-3-methylbut-2-enyl diphosphate reductase in tobacco BY-2 cell cultures. *FEBS Lett*. 2010;584:129–34 19903472 10.1016/j.febslet.2009.11.010

[38] Okada K , Kasahara H, Yamaguchi S. et al. Genetic evidence for the role of isopentenyl diphosphate isomerases in the mevalonate pathway and plant development in *Arabidopsis*. *Plant Cell Physiol*. 2008;49:604–16 18303110 10.1093/pcp/pcn032

[39] Chen HL , Li MJ, Liu CQ. et al. Enhancement of the catalytic activity of isopentenyl diphosphate isomerase (IDI) from *Saccharomyces cerevisiae* through random and site-directed mutagenesis. *Microb Cell Fact*. 2018;17:65 29712558 10.1186/s12934-018-0913-z PMC5925831

[40] Ma DM , Li G, Alejos-Gonzalez F. et al. Overexpression of a type-I isopentenyl pyrophosphate isomerase of *Artemisia annua* in the cytosol leads to high arteannuin B production and artemisinin increase. *Plant J*. 2017;91:466–79 28440881 10.1111/tpj.13583

[41] Zhao L , Zhang J, Liu T. et al. Design, synthesis, and antiviral activities of coumarin derivatives containing dithioacetal structures. *J Agric Food Chem*. 2020;68:975–81 31891504 10.1021/acs.jafc.9b06861

[42] Durbecq V , Sainz G, Oudjama Y. et al. Crystal structure of isopentenyl diphosphate: dimethylallyl diphosphate isomerase. *EMBO J*. 2001;20:1530–7 11285217 10.1093/emboj/20.7.1530 PMC145486

[43] Zhu F , Wen WW, Cheng YJ. et al. The metabolic changes that effect fruit quality during tomato fruit ripening. *Mol Hortic*. 2022;2:2 37789428 10.1186/s43897-022-00024-1 PMC10515270

[44] Nakamura A , Shimada H, Masuda T. et al. Two distinct isopentenyl diphosphate isomerases in cytosol and plastid are differentially induced by environmental stresses in tobacco. *FEBS Lett*. 2001;506:61–4 11591371 10.1016/s0014-5793(01)02870-8

[45] Zheng W , Sun F, Bartlam M. et al. The crystal structure of human isopentenyl diphosphate isomerase at 1.7 Å resolution reveals its catalytic mechanism in isoprenoid biosynthesis. *J Mol Biol*. 2007;366:1447–58 17250851 10.1016/j.jmb.2006.12.055

[46] Wouters J , Oudjama Y, Barkley SJ. et al. Catalytic mechanism of *Escherichia coli* isopentenyl diphosphate isomerase involves Cys-67, Glu-116, and Tyr-104 as suggested by crystal structures of complexes with transition state analogues and irreversible inhibitors. *J Biol Chem*. 2003;278:11903–8 12540835 10.1074/jbc.M212823200

[47] Yan N , Liu Y, Zhang H. et al. Solanesol biosynthesis in plants. *Molecules*. 2017;22:510 28333111 10.3390/molecules22040510 PMC6154334

[48] Broman KW , Wu H, Sen S. et al. R/QTL: QTL mapping in experimental crosses. *Bioinformatics*. 2003;19:889–90 12724300 10.1093/bioinformatics/btg112

[49] Deng L , Wang H, Sun CL. et al. Efficient generation of pink-fruited tomatoes using CRISPR/Cas9 system efficient generation of pink-fruited tomatoes using CRISPR/Cas9 system. *J Genet Genomics*. 2018;45:51–4 29157799 10.1016/j.jgg.2017.10.002

[50] Weigel D , Glazebrook J. Transformation of *Agrobacterium* using the freeze-thaw method. *Cold Spring Harb Protoc*. 2006;2006: pdb.prot4666-1036 10.1101/pdb.prot4666 22484682

[51] Zhao WH , Gao L, Li YH. et al. Yellow-fruited phenotype is caused by 573 bp insertion at 5′ UTR of *YFT1* allele in *yft1* mutant tomato. *Plant Sci*. 2020;300:110637 33180715 10.1016/j.plantsci.2020.110637

[52] Mao J , Zhang YC, Sang Y. et al. A role for *Arabidopsis* cryptochromes and COP1 in the regulation of stomatal opening. *Proc Natl Acad Sci USA*. 2005;102:12270–5 16093319 10.1073/pnas.0501011102 PMC1189306

[53] Nelson BK , Cai X, Nebenführ A. A multicolored set of *in vivo* organelle markers for co-localization studies in *Arabidopsis* and other plants. *Plant J*. 2007;51:1126–36 17666025 10.1111/j.1365-313X.2007.03212.x

[54] Voinnet O , Rivas S, Mestre P. et al. Retracted: an enhanced transient expression system in plants based on suppression of gene silencing by the p19 protein of tomato bushy stunt virus. *Plant J*. 2003;33:949–56 12609035 10.1046/j.1365-313x.2003.01676.x

[55] Kilambi HV , Kumar R, Sharma R. et al. Chromoplast-specific carotenoid associated protein appears to be important for enhanced accumulation of carotenoids in *hp1* tomato fruits. *Plant Physiol*. 2013;161:2085–101 23400702 10.1104/pp.112.212191 PMC3613478

[56] Bradford MM . A rapid and sensitive method for the quantitation of microgram quantities of protein utilizing the principle of protein-dye binding. *Anal Biochem*. 1976;72:248–54 942051 10.1016/0003-2697(76)90527-3

[57] Li ZD , Ji J, Wang G. et al. Cloning and heterologous expression of isopentenyl diphosphate isomerase gene from *Lycium chinense*. *J Plant Biochem Biotechnol*. 2016;25:40–8

[58] Rampogu S , Gajula RG, Lee G. et al. Unravelling the therapeutic potential of marine drugs as SARS-CoV-2 inhibitors: an insight from essential dynamics and free energy landscape. *Comput Biol Med*. 2021;135:104525 34252682 10.1016/j.compbiomed.2021.104525 PMC8164349

[59] Wu GS , Robertson DH, Brooks CL. et al. Detailed analysis of grid-based molecular docking: a case study of CDOCKER – a CHARMm-based MD docking algorithm. *J Comput Chem*. 2003;24:1549–62 12925999 10.1002/jcc.10306

[60] Rampogu S , Zeb A, Baek A. et al. Discovery of potential plant-derived peptide deformylase (PDF) inhibitors for multidrug-resistant bacteria using computational studies. *J Clin Med*. 2018;7:563 30563019 10.3390/jcm7120563 PMC6306950

