# Supplementary material for: Mutation of YFT3, an isomerase in the isoprenoid biosynthetic pathway, impairs its catalytic activity and carotenoid accumulation in tomato fruit
Source: Hortic Res. 2024 Jul 24;11(9):uhae202. doi: 10.1093/hr/uhae202 (PMC11415240; doi:10.1093/hr/uhae202)
Supplement: Web_Material_uhae202 [file web_material_uhae202.zip › Supplemental Figures 2024-06-14.pdf]

## Supplementary data

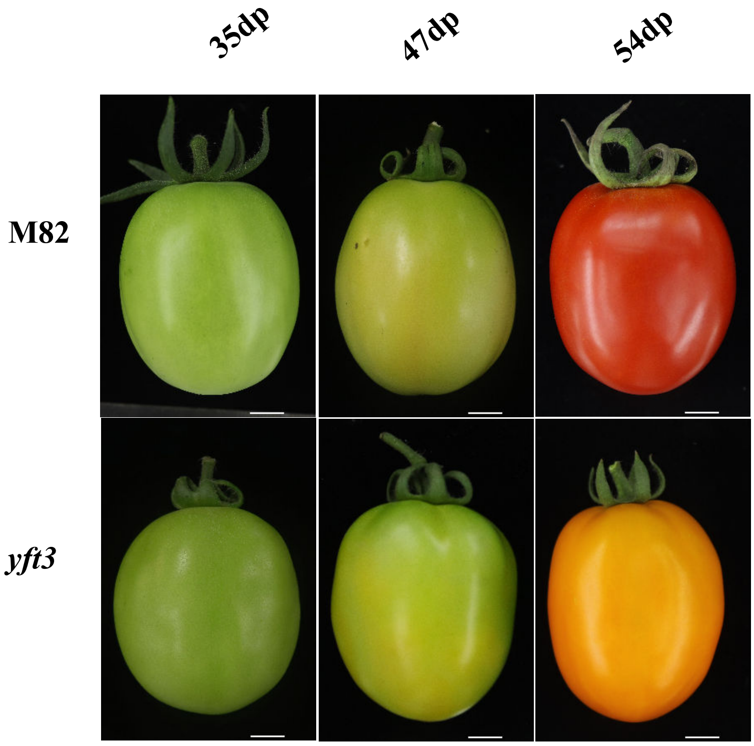


**Supplementary Fig. S1 M82 and *yft3* fruit colors at different developmental stages (35 dpa, 47 dpa and 54 dpa), bars=1.0cm**

**M82**, wild type (WT) tomato line (*Solanum lycopersicum*) used in the present study; ***yft3***, a tomato mutant, *e9292*, which was created from M82 mutagenisis by ethyl methyl sulfonate treatment; **dpa**, days post anthesis, and **35dpa, 47dpa and 54dpa** corresponding to different developmental stages of mature green (MG), breaker (BR), and red/yellow ripening (RR or YR), respectively.

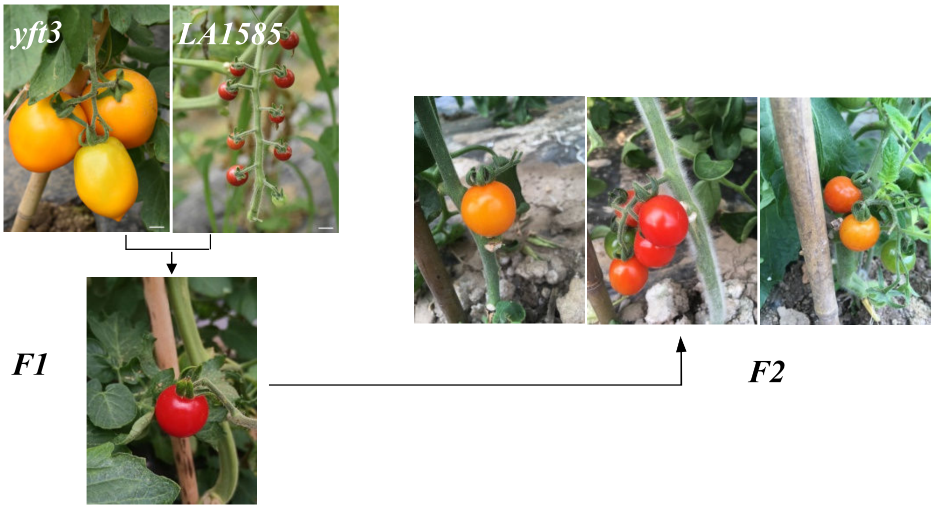


**Supplementary Fig. S2 A *yft3* mutant population was created by crossing *yft3* and LA1585 (*S. pimpinellifolium*).**

As *yft3* mutant (*S.lycopersicum*) used as pistil parent, which was crossed with LA1585(*S. pimpinellifolium*) to create mutant populations of different generations(F1 and F2), which were used to confirm genetic basis of fruit color phenotype in *yft3* mutant tomato, and to map and identify the target gene.

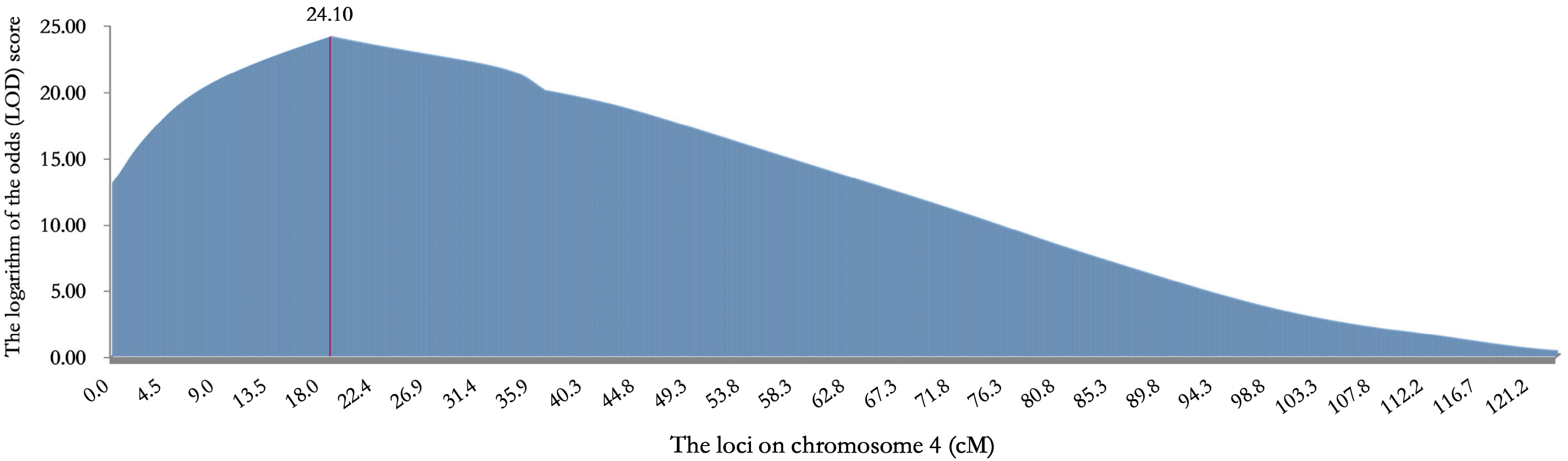


**Supplementary Fig. S3 Logarithm of the odds (LOD) score of different genes on chromosome 4 calculated from the $F_2$ population of *yft3* × LA1585 (*S. pimpinellifolium*)**

Based on the fruit color and genotypes derived from 116 plants in the F2 generation(*yft3*×LA1585), the logarithm of the odds (LOD) scores were displayed on all 12 chromosomes in tomato by R/qtl analysis, and the maximal LOD score (24.10) was identified on the 4th chromosome.

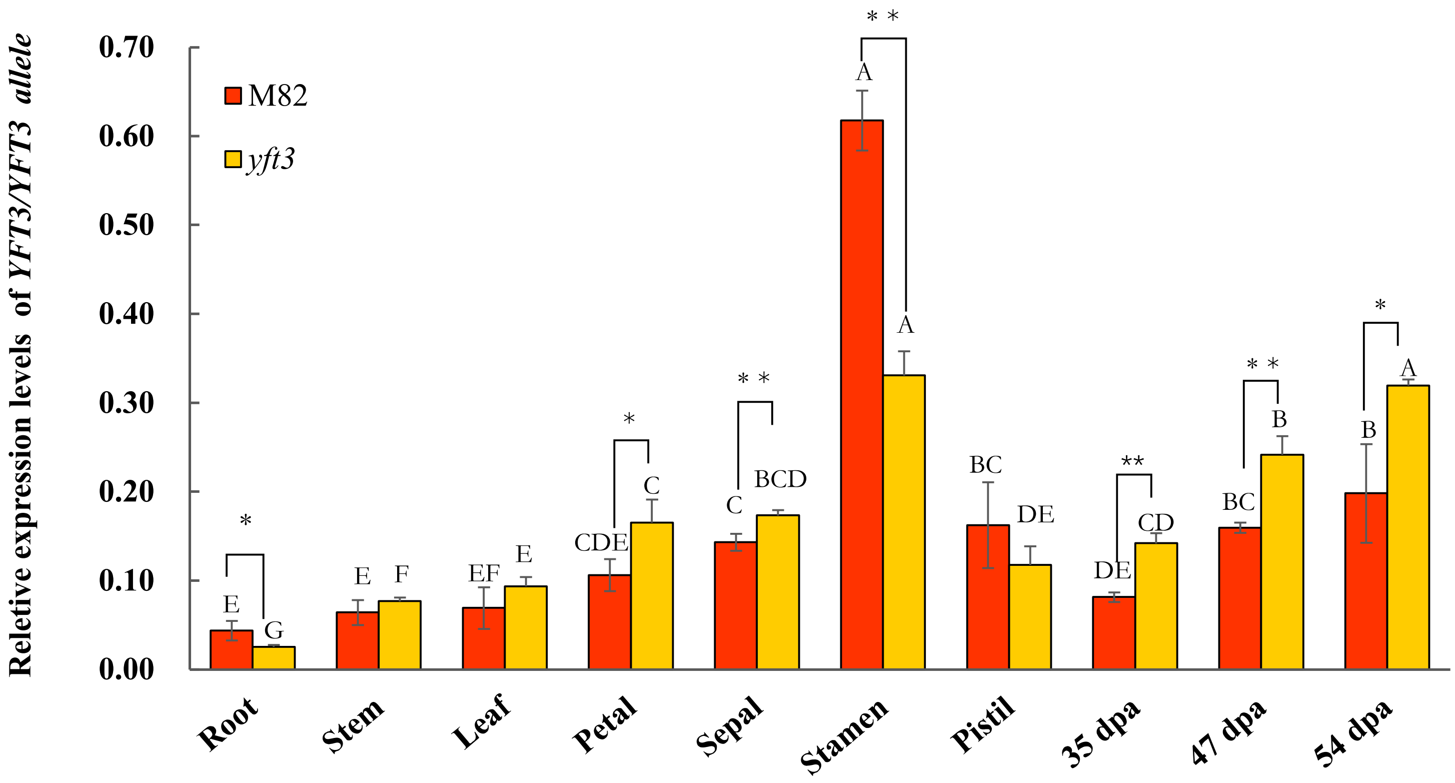


**Supplementary Fig. S4 Expression levels of *YFT3* /*YFT3* allele in different organs of M82 and *yft3*.**

**M82**, wild type (WT) tomato line (*Solanum lycopersicum*); ***yft3***, a tomato mutant induced from M82 tomato by ethyl methyl sulfonate treatment. The data are presented as mean ± SD (n=3). **Capital letters** indicate statistical significance at P<0.01 among organs of M82 and *yft3* as determined by a Duncan's test. ***** and ****** indicate statistical significance in the same organ between M82 and *yft3* at P<0.05 and P<0.01 as determined by T-test. 35dpa, 47dpa, and 54dpa indicate the different developmental stages (MG, BR, and RR/YR).

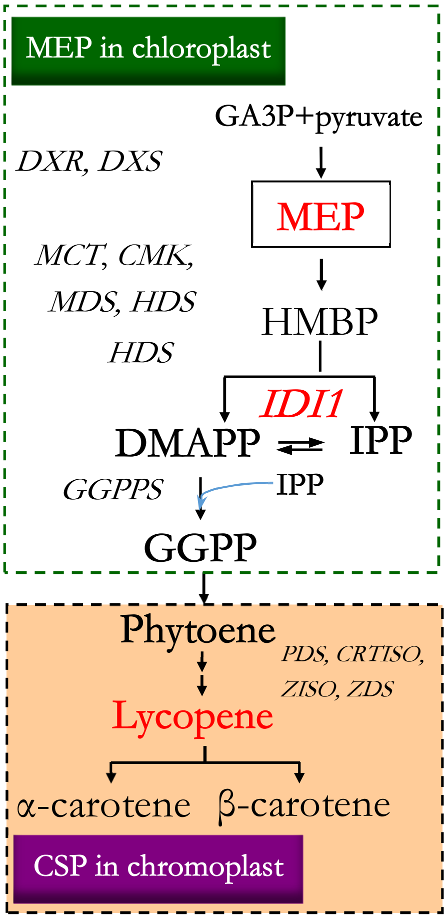


**Supplementary Fig. S5 The two successive biosynthesis carotenoid pathways MEP (2-C-methyl-D-erythritol-4-phosphate) and CSP (carotenoid synthesis pathway)**

**MEP** pathway. **Components**: **GA3P,** D-Glyceraldehyde 3-phosphate**; Pyruvate**,1-Deoxy-D-xylulose 5-phosphate**; MEP,** 2-*C*-Methyl-D-erythritol 4-phosphate**; HMBPP,** 4-Hydroxy-3-methylbut-2-enyl-diphosphate**; IPP**, Isopentenyl diphosphate, C5; **DMAPP**, dimethylallyl diphosphate, C5; **GGPP**, Geranylgeranyl diphosphate, C20. **Enzymes/genes**: **DXS**, 1-Deoxy-D-xylulose 5-phosphate synthase; **DXR**, 1-Deoxy-D-xylulose 5-phosphate reductoisomerase; **MCT**, 2-*C*-methyl-D-erythritol 4-phosphate cytidylyltransferase; **CMK**, 4-(cytidine 5-diphospho)-2-*C*-methyl-D-erythritol kinase; **MDS**, 2-*C*-methyl-D-erythritol 2,4-cyclodiphosphate synthase; **HDS**, 4-Hydroxy-3-methylbut-2-enyl-diphosphate synthase; **HDR**, 4-Hydroxy-3-methylbut-2- enyl diphosphate reductase; **IDI**, Isopentenyl diphosphate *Δ*-isomerase; **GGPPS**, Geranylgeranyl diphosphate synthase.
**CSP** (carotenoid synthesis pathway). **Enzymes/genes: CCDs**, carotenoid cleavage dioxygenases; **CHYB**, β-ring carotene hydroxylase; **CHYE**, ε-ring carotene hydroxylase; **CRTISO**, carotene isomerase; **LCYB/CYCB**, lycopene β-cyclase; **LCYE**, lycopene ε-cyclase; **MCT**, 4-diphosphocytidyl-2C-methyl-D-erythritol synthase; **NCEDs**, 9-cis-epoxycarotenoid dioxygenases; **NSY**, neoxanthin synthase; **PDS**, phytoene desaturase; **PSY**, phytoene synthase; **ZDS**, ζ-carotene desaturase; **ZEP**, zeaxanthin epoxidase; **ZISO**, ζ-carotene isomerase. **Green dashed box**, MEP pathway; **Black dashed box**, CSP.

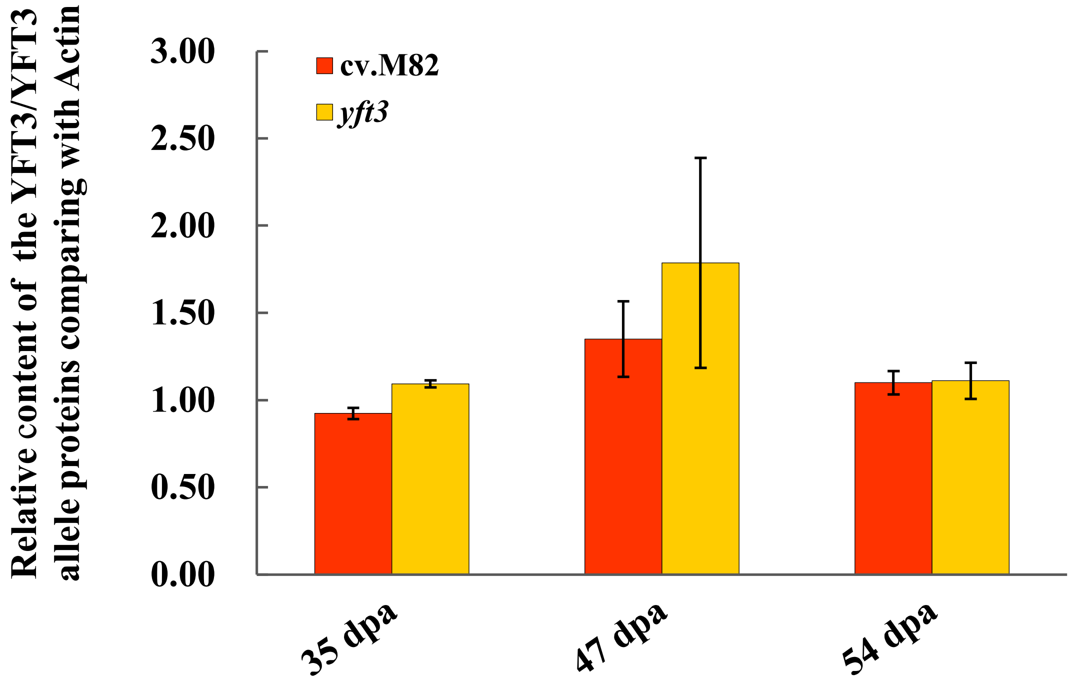


**Supplementary Fig. S6 Relative content of YFT3 and YFT3 allele proteins compared with Actin in M82 and *yft3* tomato fruits at different developmental stage**

The relative contents of the YFT3 and YFT3 allele proteins were normalized using Actin as an internal reference protein in this study.
