# Supplementary material for: Mutation of YFT3, an isomerase in the isoprenoid biosynthetic pathway, impairs its catalytic activity and carotenoid accumulation in tomato fruit
Source: Hortic Res. 2024 Jul 24;11(9):uhae202. doi: 10.1093/hr/uhae202 (PMC11415240; doi:10.1093/hr/uhae202)
Supplement: Web_Material_uhae202 [file web_material_uhae202.zip › Supplemental tables 2024-06-14.pdf]

Supplementary data

Supplementary Table S1 CAPS and dCAPS markers used for gene mapping*

Marker ID	Physical position	Length	Primer sequence (5'-3')	Marker category	Restriction enzyme	Chromosome	Application
C2_At5g51970	SL2.50ch01:1097088..1098015	928 bp	acgcgagttttgactgtgctgg<br>tcttcttgagagaatccaaacctgtg	CAPS	*Xba* I	1	primary mapping
C2_At3g08030	SL2.50ch01:82482380..82483201	822 bp	aggcttcaatttctcagacaattcc<br>ttcaaaaccagcatttttaaccaag	CAPS	*Hinc* II	1	primary mapping
C2_At2g38730	SL2.50ch01:87604698..87605239	542 bp	agcggaccaaacactaatggatg<br>agccacattctcaatcttcctgac	CAPS	*Alu* I	1	primary mapping
C2_At2g16920	SL2.50ch01:97872062..97873113	1052 bp	tgcaagacaatgatgtatctgatgagg<br>tcctaaagtgctctctgataagctcttc	Indel		1	primary mapping
T1117	SL2.50ch02:35214141..35214484	344 bp	caactccgtctttgtcagcc<br>tgctttgtcgtctgcaccct	CAPS	*Bgl* II	2	primary mapping
C2_At4g35560	SL2.50ch02:46349593..46349971	379 bp	agttcaacacgtgtagcttgcatgg<br>aacagatcaatactaatggttgctttg	CAPS	*EcoR* I	2	primary mapping
T1480	SL2.50ch02:50645678..50647141	1464 bp	accaccttggatgaataccg<br>tgcaacagcttttccctctc	CAPS	*Alu* I	2	primary mapping
TG585	SL2.50ch03:1238732..1239222	491 bp	tggaaagccagacacacaga<br>caggggtatcagtaggcagtg	CAPS	*Dra* I	3	primary mapping
30M2	SL2.50ch03:29643129..29643844	716 bp	aagttagagtcctatgtgggaagat<br>ggggtgttgttaggatgatgaatat	CAPS	*Afl* II	3	primary mapping
TG134	SL2.50ch03:64914334..64914531	198 bp	aattccaagtcccggttag<br>aatgttactgtaggcgaatgtg	CAPS	*Taq* I	3	primary mapping
C2_At3g17040	SL2.50ch04:2593402..2593876	475 bp	tggggttggatggagtggaaag	CAPS	*Bgl* II	4	primary mapping

C2_At3g62940	SL2.50ch04:51435545..51436456	912 bp	agtagaggttacgaatttcctctgc<br>acccgcgaggagatgctttctagg<br>tctgctctctggctgcctcttgttg	CAPS	*Ssp*I	4	primary mapping
M256	SL2.50ch4:52561306..52562558	1253 bp	gcgcaactaagatgaggttatg<br>ctccatcttggcttgactgattac	CAPS	*Bgl*II	4	Fine mapping
M865	SL2.50ch4:58657671..58659120	1450 bp	gaacggcagggagatggaag<br>gaaaatcgtcatggtgcttagagac	CAPS	*DraI*	4	Fine mapping
311M	SL2.50ch4:53111372..53112523	1152 bp	ccaggtttaagtcttctactgtccg<br>ggcactagttaagttggtagatggt	CAPS	*Afl*II	4	Fine mapping
404M	SL2.50ch4:54046020..54047002	983 bp	ggtaggaagcggtatcaccatttggg<br>cgtactaagtattactactatggttgcgaagag	CAPS	*Hpy188*I	4	Fine mapping
428M	SL2.50ch4:54284003..54285299	1297 bp	tgatcttgaacagcctgcttga<br>ctgccatagtttgactgggtaa	CAPS	*Ase*I	4	Fine mapping
542M	SL2.50ch4:55422376..55423853	1478 bp	ccagcgccaaggagcatacc<br>ctgctcgtgtccccaagaag	CAPS	*EcoR*V	4	Fine mapping
780M	SL2.50ch4:57801839..57802683	845 bp	tacgctccaaatgtctttccta<br>ccttggagctctcgtcttggt	CAPS	*Afl*II	4	Fine mapping
C2_At1g10030	SL2.50ch04:62086451..62088182	1732 bp	agctgttaggatggtggttaatgc<br>actctgtcaagaaatgaccgaaggc	CAPS	*EcoR*V	4	primary mapping
C2_At3g16150	SL2.50ch04:63185695..63186066	372 bp	aagcaactccttactcgttgcc<br>tggagataccagaaacggcg	CAPS	*Hinf*I	4	primary mapping
CT173	SL2.50ch04:65017593..65019030	1438 bp	tagaaagggcttataaactggga<br>gtaagagacatttcgagcattc	CAPS	*Taq*I	4	primary mapping
TG441	SL2.50ch05:1948922..1949367	446 bp	tggtatggtaccacgacaaag<br>ttttcaggtcgaggataccg	CAPS	*Taq* I	5	primary mapping
C2_At1g30110	SL2.50ch05:2430835..2431490	656 bp	aatgtacccggagcatggcaaatg	CAPS	*Ase* I	5	primary mapping

			tgggaagtcatacgtcaaccattg				
C2_At2g01275	SL2.50ch05:2757660..2758912	1253 bp	tgccgctgattgctcttcccag<br>tgccgtataaccaggcctaaaag	Indel		5	primary mapping
C2_At1g26670	SL2.50ch05:6158394..6159285	892 bp	aaaatggatctcgaggcaagaag<br>aagcgttccctttgacttgcagaag	CAPS	*Dra*I	5	primary mapping
C2_At2g01720	SL2.50ch05:65529493..65529984	492 bp	acaaattggtacatgctggtgctc<br>tggcctgttagactgatattcaac	CAPS	*Dra*I	5	primary mapping
T0774	SL2.50ch06:33536970..33537905	936 bp	agcaaagtatttcccgtctc<br>tcctcaactggctaattctca	CAPS	*Hinf*I	6	primary mapping
C2_At4g10030	SL2.50ch06:40285377..40286959	1583 bp	tcgcgtcagctgctcttgatgtc<br>tttcctccaaagctgtgaccaac	CAPS	*Hinf* I	6	primary mapping
C2_At1g24360	SL2.50ch06:44301895..44302661	767 bp	tccggttgttattgtcactggagc<br>tggaaacttcttctgcctcctttg	dCAPS	*Taq*I	6	primary mapping
C2_At2g24270	SL2.50ch07:297981..298962	982 bp	tggaaatgaaaggaccaaatactg<br>aggagcaatcttggagacggc	CAPS	*Hin*f I	7	primary mapping
C2_At2g20860	SL2.50ch07:62834193..62835515	1333 bp	attgaagccacatatactcatagaagc<br>tccagattttgcaactttctctacac	CAPS	*Dra*I	7	primary mapping
TG302	SL2.50ch08:58653183..58653941	759 bp	ctctccgggtggctattaca<br>tcttgggactcctccttttct	CAPS	*Alu* I	8	primary mapping
C2_At4g11560	SL2.50ch08:64169481..64170987	1507 bp	tccagaaggacaacttcagaagcc<br>tgtttaatggtatgaagtgcaccacac	CAPS	*Dpn* II	8	primary mapping
C2_At3g27530	SL2.50ch08:64483969..64485260	1292 bp	agcatccaatgcttgcatcagtatttg<br>tcccaccagttctggccagatcctc	CAPS	*Dra* I	8	primary mapping
TG294	SL2.50ch08:65747409..65748309	901 bp	attggctgcaatgatggatt<br>ctaagcaggacggccatcta	CAPS	*Xmn* I	8	primary mapping
T1641	SL2.50ch09:1095924..1096332	409 bp	ccaacttctagataaagtac	CAPS	*Xho* I	9	primary mapping

cLEX-3-N24	SL2.50ch09:750164..750671	508 bp	cagctcttgggcaactctttg<br>cgaacttcaattcagtacga	dCAPS	*Hinf*I	9	primary mapping
T1190	SL2.50ch09:69684800..69686689	1890 bp	gaggatgtacgcgatcagcc<br>gcgttctcgttactggtgct	CAPS	*Rsa* I	9	primary mapping
T0156	SL2.50ch09:70745881..70746880	1000 bp	gttgcatggttgacatcagg<br>gcggttgattcacatcgtaa	CAPS	*Dpn* II	9	primary mapping
C2_At5g02020	SL2.50ch09:4795100..4795638	539 bp	cctgtagcacccaaaggatg<br>gctacatcatcttgcaaggagc	CAPS	*EcoR* I	9	primary mapping
C2_At4g30220	SL2.50ch10:62463379..62463576	198 bp	ctataagctttccgcttgtggtg<br>actggaaagcctgtgatggtaaagc	CAPS	*Hinf*I	10	primary mapping
TG233	SL2.50ch10:65356898..65357387	490 bp	tgcaagttcatatatgaatccacagagac<br>catgcctttttcttgggatg	CAPS	*Hinc* II	10	primary mapping
C2_At5g60990	SL2.50ch10:1856461..1857851	1391 bp	tggaacccctttaactgtgc<br>tgatacactgaagcagcagtatcg	CAPS	*HinfI*	10	primary mapping
TG497	SL2.50ch11:713458..714346	889 bp	agccagaagacgagttgcatcac<br>cggagagtgaagatgcattg	CAPS	*Taq*I	11	primary mapping
C2_At3g52220	SL2.50ch11:1356074..1356351	278 bp	aagttccagagggagcacaa<br>tgctcgggtggatggtcttgg	CAPS	*Mse* I	11	primary mapping
C2_At4g22260	SL2.50ch11:4940984..4941704	721 bp	tgatggtgaacttggttcttccc<br>tcctctaacggtctagagaaatggg	CAPS	*Hinf*I	11	primary mapping
T0302	SL2.50ch11:54794676..54795467	792 bp	aggaactcttgcaattgtttccagaac<br>tggctcatcctgaagctgatagcgc	CAPS	*Msp*I	11	primary mapping
TG393	SL2.50ch11:56166010..56166744	735 bp	agtgtacatccttgccattgact<br>tggatttgattagccgaagg	CAPS	*Dpn*II	11	primary mapping
TG180	SL2.50ch12:160631..161631	1001 bp	ccaagaatcccagaaggaga<br>tctcagtggactaaggggtca	CAPS	*Dra* I	12	primary mapping

cLET-8-K4	SL2.50ch12:3203617..3204849	1233 bp	tcacagcagacatgtcggac<br>cactttgtggcaatcgacat<br>tgccttatgccaaacagaaa	CAPS	*Taq*I	12	primary mapping
C2_At1g48300	SL2.50ch12:66232963..66233867	905 bp	aagaagatgaaattacttaagggtttg<br>tttagtgttgcattctcaagtgctcg	CAPS	*Hind*III	12	primary mapping

* CAPS, cleaved amplified polymorphic sequences; dCPAS, derived cleaved amplified polymorphic sequence; Indel, insertion-deletion. All CAPS and dCAPS markers in the table were chosen and designed based on differences in the DNA sequences between *yft3* (*S.lycopersicum*) and LA 1585 (*S.pinpimellifolium*), and spanned all 12 chromosomes. In particular, the makers listed in lines with pale green highlights of the table were newly developed from the primary mapping region for fine mapping of the target gene.

Supplementary Table S2 Candidate genes in the fine mapping region between the CAPS M404 and M428 markers on chromosome 4*

Gene ID	Description	Position
Solyc04g05632	Zinc finger	54,066,672..54,067,793
Solyc04g05633	AIG2-like family protein	54,0720,65..54,072,580
Solyc04g05634	Tyrosine phosphatase family protein	54,089,361..54,091,534
Solyc04g05635	Zinc finger (C2H2 type) family protein	54,112,402..54,113,719
Solyc04g05636	WRKY transcription factor 36	54,133,842..54,135,811
Solyc04g05637	Pentatricopeptide repeat-containing protein	54,153,793..54,158,336
Solyc04g05638	Seipin-1	54,160,438..54,164,277
Solyc04g05639	Isopentenyl diphosphate Delta-isomerase	54,164,138..54,167,905
Solyc04g05641	Protein CHROMATIN REMODELING 25	54,181,225..54,194,900
Solyc04g05642	Peroxidase	54,198,332..54,199,541
Solyc04g05644	Cyclopropane-fatty-acyl-phospholipid synthase	54,211,820..54,214,299
Solyc04g05645	Cyclopropane-fatty-acyl-phospholipid synthase	54,214,940..54,221,299
Solyc04g05647	BZIP transcription factor 17	54,257,077..54,258,156
Solyc04g05648	Unknown protein	54,263,797..54,264,760
Solyc04g05649	TGACG-sequence-specific DNA-binding	54,282,053..54,282,721

* The highlighted line in pale green in the table lists the functions and physical positions of the candidate gene *SlIDI1* of *YFT3*.

Supplementary Table S3 Primers used in this study

Primer ID	Primer sequence（5’ to 3’）	Application
*35S-CDS-Bam*H I	cacgggggactctagaggatccatgagaggaattgatgggaaca	CDS of *YFT3*
35S-CDS-Sac I	gaacgatcggggaaattcgagctcttaagtcaatttgtggatgg	
cri-YFT3-F	atatatggtctcgtttgcggaaacggcaacggagaggttttagagc	Amplification of the dual target fragment
cri-YFT3-R	attattggtctcgaaactcctcacctgcatctgcttcaaactacact	
$pTX\text{-}F_W$	agcggataacaatttcacacagga	Check for CRISPR-Cas 9 lines
$pTX\text{-}R_V$	gcaggcatgcaagcttattgg	
T1-CRI-F	gaacataaacaaatgagggatagg	Check for target 1
T1-CRI-R	cttaggttggtgttttgttgagg	
T2-CRI-F	acagcattgagggtgttttgac	Check for target 2
T2-CRI-R	ctgaaatggctcaaaggacg	
*YFT3-GFP-Bam*H Ⅰ	ctcaagcttggatccatgagaggaattgatgggaac	Subcellular localization
YFT3-GFP-Spe Ⅰ	gctcaccatactagtagtcaatttgtggatggttttc	
*YFT3-ex-Bam*H I	gcaaatgggtcgcggatccatgagaggaattgatgggaacaag	Prokaryotic expression of recombinant vector
YFT3-ex-Sac I	caagcttgtcgacggagctcagtcaatttgtggatggttttcatatc	
qYFT3-F	gagaggaattgatgggaacaagccg	RT-qPCR for *IDI1*
qYFT3-R	ccaacaacatggtcattctcatccacc	
qDXS-F	aagaggaaatgggatcggtgta	RT-qPCR for *DXS*
qDXS-R	agccactctctccccctcaa	
qDXR-F	cttaggcgcattatattaactgcat	RT-qPCR for *DXR*
qDXR-R	gtggcagaatcaacagtaatctttt	
qHDR-F	cgggatgcctaaggctaaactcc	RT-qPCR for *HDR*
qHDR-R	ctggtcagattcttccg	
qPSY1-F	tggcccaaacgcatcatata	RT-qPCR for *PSY1*
qPSY1-R	caccatcgagcatgtcaaatg	
qCRTISO-F	ttttggcggaatcaactacc	RT- qPCR for *CRTISO*
qCRTISO-R	gaaagcttcgctcccacag	
qCYCB-F	tgttattgaggaagagaaatgtgtgat	RT-qPCR for *CYCB*
qCYCB-R	tcccaccaatagccataacatttt	

qCYP707A2-F	tcgaaaaaggatacaattcgatgcc	RT-qPCR for *CYP707A2*
qCYP707A2-R	ctgcaatttgttcgtcagtgagtcc	
qNCED-F	aggcaacagtgaaacttccatcaag	RT-qPCR for *NCED*
qNCED-R	tccattaaagaggatattaccggggac	
Actin-F	ttgctgaccgtatgagcaag	RT-qPCR for *ACTIN*
Actin-R	ggacaatggatggaccagac	

Supplementary Table S4 Molecular docking of YFT3 and YFT3 allele proteins with IPP and DMAPP *

	AA residue	Bond categories	Bond length(Å)		AA residue	Bond categories	Bond length(Å)
**YFT3-IPP**	Cys157	CC double bond of the alkylate	5.04	**YFT3-DMAPP**	His110	alkyl	4.42
	Ser158	hydrogen bonds	2.80		His122	carbon hydrogen bond	2.81
	Tyr207	hydrogen bonds	2.90		Ser126	hydrogen bond	2.75
	Trp269	Pi-Anion	3.62		Arg141	Pi-anion	5.06
**YFT3 allele-IPP**	His110	CC double bonds of the Pi-Alkyl	5.26		Ser158	hydrogen bond, carbon hydrogen bond	2.84, 2.10
	Arg141	hydrogen-carbon bond, attractive charges	2.79, 3.62, 3.38, and 4.68		Tyr207	hydrogen bond	2.39
	Lys145	hydrogen bond, attractive charge	2.51, 4.91		Glu217	hydrogen bond	2.50
	Cys156	hydrogen bond, hydrogen-carbon bond	2.39, 2.62		Glu219	hydrogen bond	1.99

	Cys157	CC double bonds of Pi-Alkyl, hydrogen bond	4.83, 1.99, 2.72		Trp269	Pi-anion	2.96
	Ser158	hydrogen-carbon bond	2.96	**YFT3 allele-DMAPP**	Arg141	attractive charge	4.57
	Lys182	attractive charge	5.09		Cys156	alkyl reaction	4.48
	Glu217	hydrogen-carbon bond	2.49		Tyr207	hydrongen bond	2.94
					Trp269	Pi-Anion	3.08
