# Supplementary material for: Mutation of YFT3, an isomerase in the isoprenoid biosynthetic pathway, impairs its catalytic activity and carotenoid accumulation in tomato fruit
Source: Hortic Res. 2024 Jul 24;11(9):uhae202. doi: 10.1093/hr/uhae202 (PMC11415240; doi:10.1093/hr/uhae202)
Supplement: Web_Material_uhae202 [file web_material_uhae202.zip › TOC-03.pdf]

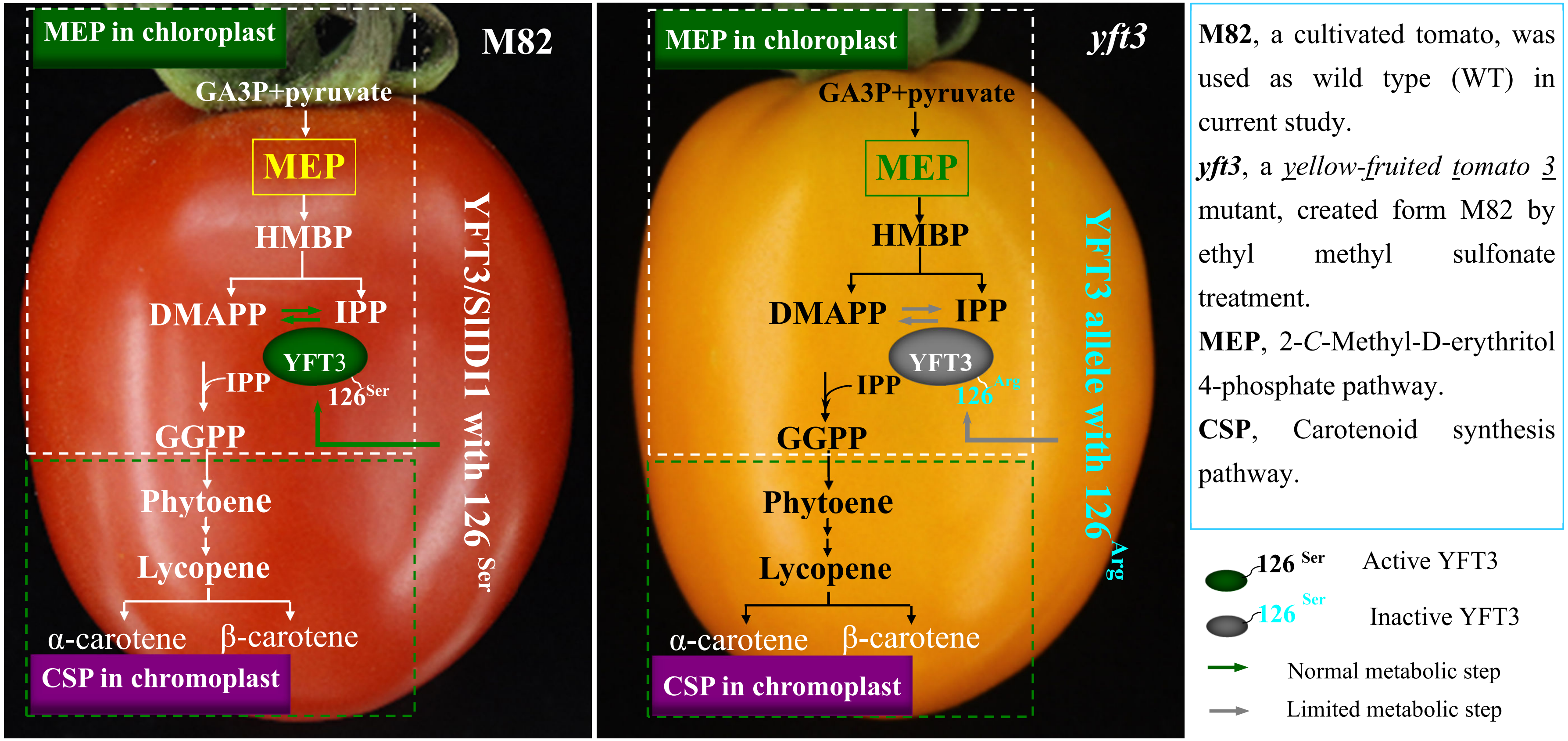

TOC: A Ser126Arg substitution in *yft3* tomato mutant alters fruit color due to serious impairment of YFT3 allele catalytic activity in the isoprenoid biosynthetic pathway.
